# Impact of educational interventions on adolescent attitudes and knowledge regarding vaccination: A pilot study

**DOI:** 10.1371/journal.pone.0190984

**Published:** 2018-01-19

**Authors:** Kate Carolan, Joanna Verran, Matthew Crossley, James Redfern, Nicola Whitton, Martyn Amos

**Affiliations:** 1 School of Computing, Mathematics and Digital Technology, Manchester Metropolitan University, Manchester, United Kingdom; 2 School of Healthcare Science, Manchester Metropolitan University, Manchester, United Kingdom; 3 Faculty of Education, Manchester Metropolitan University, Manchester, United Kingdom; The Chinese University of Hong Kong, HONG KONG

## Abstract

**Background:**

Current immunisation levels in England currently fall slightly below the threshold recommended by the World Health Organization, and the three-year trend for vaccination uptake is downwards. Attitudes towards vaccination can affect future decisions on whether or not to vaccinate, and this can have significant public health implications. Interventions can impact future vaccination decisions, and these interventions can take several forms. Relatively little work has been reported on the use of vaccination interventions in young people, who form the next generation of individuals likely to make vaccination decisions.

**Method:**

We investigated the impact of two different types of educational intervention on attitudes towards vaccination in young people in England. A cohort of young people (n = 63) was recruited via a local school. This group was divided into three sub-groups; one (n = 21) received a presentation-based intervention, one (n = 26) received an interactive simulation-based intervention, and the third (n = 16) received no intervention. Participants supplied information on (1) their attitudes towards vaccination, and (2) their information needs and views on personal choice concerning vaccination, at three time points: immediately before and after the intervention, and after six months.

**Results:**

Neither intervention had a significant effect on participants’ attitudes towards vaccination. However, the group receiving the presentation-based intervention saw a sustained uplift in confidence about information needs, which was not observed in the simulation-based intervention group.

**Discussion:**

Our findings with young people are consistent with previous work on vaccination interventions aimed at adults, which have shown limited effectiveness, and which can actually reduce intention to vaccinate. Our findings on the most effective mode of delivery for the intervention should inform future discussion in the growing “games for health” domain, which proposes the use of interactive digital resources in healthcare education.

## Introduction

*Vaccination* [1] is a process whereby an individual may become artificially immunised against an infectious disease. Deliberate introduction of antigens (i.e., the vaccine) stimulates the body to produce antibodies, which allow it to fight off future exposure to a disease. *Herd immunity* is the effect produced by a significant proportion of a population being immunised against an infectious disease [2]. Immunocompromised individuals cannot receive vaccines containing live or attenuated cells, and receiving the vaccine could lead to their contracting an active infection. Herd immunity is therefore important to both the eradication and containment of serious infectious diseases, and to the protection of those who cannot be vaccinated, by creating a “barrier” of immunised people. However, herd immunity can be compromised if the proportion of vaccinated individuals in a population drops below a critical threshold.

Current guidance from the European Region of the World Health Organization (WHO) recommends that at least 95% of children be immunised against specific diseases such as diphtheria, tetanus, pertussis, polio, Hib, measles, mumps and rubella [3]. The latest available National Health Service statistics for England show that in 2015-16 93.6% of children reaching their first birthday had completed their primary immunisation courses against diptheria, tetanus, pertussis, polio, and Hib (compared with 94.2% in 2014-15, and 94.3% in 2013-14), and coverage of the first dose of the measles, mumps and rubella (MMR) vaccine for children reaching their second birthday stood at 91.9% in the same period (compared with 92.3% in 2014-15, and 92.7% in 2013-14) [4].

Current immunisation levels in England therefore fall slightly below the WHO threshold, and the three-year trend for vaccination is downwards. The issue of *vaccination resistance/refusal* [5–7] in *parents* is complex and multi-factorial [8–10], and falls outside the scope of our study. However, recent arguments [11, 12] suggest that public health efforts to address issues of *vaccine hesitancy* [6, 13, 14] (as opposed to active *resistance*) may prove beneficial in terms of maintaining coverage. Moreover, some researchers have argued [15] that efforts in addressing vaccination hesitancy should become more focussed on *children and young people*, for two main reasons: (1) there exists recent evidence that interventions in *adults* aimed at improving vaccination rates or correcting myths about vaccines can actually be *counter-productive*, and lead to further entrenchment of anti-vaccination positions (the so-called “backfire effect”) [16, 17]; (2) Given that attitudes towards vaccination seem to be firmly-held by adulthood, if we assume that beliefs are often formed *during childhood and early adolescence*, then an opportunity exists to strengthen positive messages about vaccination through school-based educational programmes, which will hopefully influence young people’s future vaccination decisions about their own children.

Some community medicine researchers advocate the use of *games* and other digital resources in school curricula dealing with vaccination [15], but no evaluation studies have yet been performed to assess their effectiveness in terms of improving either educational or attitudinal outcomes in young people. Our study addresses precisely this gap in the literature. We assessed both digital simulation-based and traditional educational interventions with young people to assess whether or not these can affect their attitudes towards vaccination or their level of confidence in their knowledge of vaccination.

## Methodology

We took a mixed quantitative/qualitative data collection approach. This was necessary because attitudes are inherently multi-faceted [10, 18, 19]. The *Health Belief Model* (HBM) [20] underpinned the development of the interview schedule and discussion questions, as this has proved particularly effective in establishing the impact of attitudes and beliefs on behavioural intentions (and in the context of vaccination) [21]. The HBM focuses on understanding attitudes towards a health topic, by investigating the impact of “concepts” on health beliefs, including perceived *benefits* of an action such as vaccination, perceived *barriers* to the action, perceived *susceptibility and severity* (e.g. to a disease), and *cues to action* (e.g. a letter from a doctor) [20]. Several studies have used the HBM in an exploratory way [22–25]; this study used the HBM to explore the attitudes of teenagers towards vaccination during the initial research stages, using interviews (n = 14). In addition, The HBM underpinned the development of the initial interview schedule and discussion questions, as this has proved particularly effective in establishing the impact of attitudes and beliefs on behavioural intentions (and in the context of vaccination) [21]. In the next Section, we describe, specifically, how the study was influenced by the HBM.

### Study design

Prior to the main study, we performed a literature search for questionnaires exploring attitudes towards vaccination. However, none of these were found to be suitable for our purposes, for two reasons: (1) they focussed solely on adults, and/or (2) they focussed on a specific vaccine (e.g., HPV, MMR) [26–28]. For these reasons, we developed our own questionnaire (the design of which was guided by those found in the literature).

The development of the attitudinal survey proceeded over several stages. These encompassed in-depth interviews, selection of survey items, selection of a scale, validation of the questionnaire, and the use of statistical analysis to refine the survey into an eight-item questionnaire. We conducted in-depth interviews conducted with local teenagers (n = 14) to explore the *range of attitudes* towards vaccination (these individuals were not part of the main trial). We designed an interview schedule (S1 Text) to explore the full range of attitudes towards vaccination in teenagers. The interview schedule used open questions, and was semi-structured, with prompts for each question. The interview schedule was reviewed by experts in Microbiology and Education research to ensure that the questions were not leading, and used introductory questions to “settle” participants and ensure they were at ease before the main body of the interview. The interview schedule was designed around the following concepts, which are related to the Health Belief Model:

- Perceived susceptibility to infectious diseases included in the immunisation schedule
- Perceived seriousness of vaccine-preventable infectious diseases
- Perceived benefits of vaccination
- Perceived barriers to vaccination
- Sources of information in vaccination decisions.

An initial discussion task incorporated “role-play” and decision-making; participants were asked to imagine that they needed to decide whether or not to vaccinate their child against measles. They were provided with the “pros and cons” of vaccinating, using a “doctor’s” opinion and a “friend’s” opinion. The following discussion questions were supplied:

1. Are there any advantages of vaccination? If so, what are they? (Perceived benefits)
2. Are there any disadvantages of vaccination? If so, what are they? (Perceived barriers)
3. Why do you think some people don’t want to vaccinate? (Perceived barriers)
4. How serious do you think infectious diseases like measles are? (Perceived severity)
5. How likely do you think it is that someone could catch measles? (Perceived susceptibility)
6. Should people be encouraged to vaccinate by their doctors? (Personal choice)
7. What would make you more likely to vaccinate? (Cues to action)
8. Do you think that doctors or parents should have the most say about children’s vaccinations? (Personal choice)
9. Can you think of any other issues surrounding vaccination? (General discussion)

Interviews were then conducted to data saturation [29], and yielded six main themes that were considered to be important to the participants when considering issues surrounding vaccination: (1) trust, (2) effectiveness of vaccination, (3) safety of vaccination, (4) risk of infectious disease, (5) information needs, and (6) personal choice. The prevalence of these themes is consistent with previous research on attitudes towards vaccination [10, 18, 30], including a qualitative study of Scottish teenagers’ understanding towards and views of vaccination [19].

These themes informed the design of the *attitudinal survey*, with five questions designed for each of the six themes (in order to ensure a representative range). We used a Likert scale for the questionnaire (with responses coded 1-5), allowing each participant to receive a score corresponding to their overall attitude towards vaccination. *Face validity* [31] of questions was assessed by microbiology and education experts, and we made changes based on their feedback (for example, the questions were re-worded to make them more suitable for the age group.)

A Flesch Reading Ease analysis [32] of the draft questionnaire gave a score of 79.5, suggesting the questionnaire was suitable for 13-15 year olds. We then presented it to a focus group made up of subjects from the target age group (n = 9). Participants were asked to give feedback on the terminology used in the survey, as well as general opinions and thoughts about the survey. Four participants said that the questionnaire was readable as it was. Two questions were re-written based on feedback provided. In order to further refine the survey, we presented it to anonymous participants using online forums. We collected 46 responses, and used discriminant analysis, correlation analysis and Cronbach’s alpha to eliminate questions that gave similar types of response. The final attitudinal survey included eight items from the original 30 statements, covering four themes: *trust* (of doctors and healthcare professionals), *risk* of infectious diseases, *safety* of vaccination, and *effectiveness* of vaccination. The final set of additional questions included in the questionnaire also included six questions about information needs and personal choice. This gave a total of 14 questions in the survey.

The target age range for the study was 14-18; however, due to a lack of availability of appropriate participants, and the need for the trials to have some educational alignment with taught material (so that school teaching time was not “wasted”), only students aged 14-15 participated in the intervention study. However, the preliminary stages (interviews, focus groups, pilot trials) featured participants across the full 14-18 age range.

Participants in the intervention study were drawn from a Secondary school in North West England. Most of the participants were fifteen years old, just over half of the participants were male, the majority of participants were White British, and participants largely reported as being either Christian or non-religious (see Table 1 for a full breakdown). Participants were provided with a detailed information sheet and consent form prior to participation, parental permission was requested, and the researcher had a full DBS (Disclosure and Barring Service) check performed, allowing her to work unsupervised with children. Participants were recruited through a “gatekeeper” senior school staff member. Consent was obtained from participants prior to their involvement in the study (additionally, parental consent was obtained via the school), and participants could choose to not answer any question. Ethical approval for interviews and trials was granted through Manchester Metropolitan University’s Ethical Approval Procedure (application number SE141521).

**Table 1:** Demographic summary of study participants.

	N = 63	%
**Gender**		
Male	34	53.97
Female	29	46.03
**Age**		
14	29	46.03
15	34	53.97
**Ethnicity**		
Asian/Asian British	3	5.45
Mixed Ethnic Background	1	1.59
White British	59	93.65
**Religion**		
Christian	27	42.86
Buddhist	1	1.59
Pagan	1	1.59
None	29	46.03
Prefer not to say	5	7.94

The number of participants in our study (n = 63) is comparable to to that seen in similar recent studies, including one (n = 58) that looked at the effect of an educational intervention on human papillomavirus vaccine uptake in female students [33], and another (n = 54) that evaluated the impact of an educational intervention on students attitudes towards mental health [34]. Participants were each assigned a unique ID code to allow pre- and post-trial responses to be recorded consistently. Participants were assigned, according to their class, to either one of the two intervention groups, or to the control group.

We performed an initial survey of all participants using a questionnaire (S1 Questionnaire) in order to establish (1) their attitudes towards vaccination, (2) their confidence in their knowledge of vaccination, (3) their information needs, and (4) their views on personal choice concerning vaccination. This established *baseline* scores for each individual in order to assess the impact of the interventions.

Both intervention groups received material with the same learning objectives. Students should:

- Know what a pathogen is
- Understand the process of vaccination and how it leads to immunity
- Know what herd immunity is and how it is beneficial to a population
- Understand the background to measles and how it can be prevented by vaccination

One group (Group A) received the digital game-based resource (n = 26), and the other group (Group B) received a traditional PowerPoint lesson (n = 21). We denote the control group (n = 16) as Group C. Both groups A and B then participated in a short session (one per group), where the advantages and disadvantages of vaccination were discussed (both groups discussed the same questions), and participants completed a worksheet (S1 Worksheet) and a feedback survey. The control group attended their usual lessons while Groups A and B were receiving the interventions, and did not participate in a discussion. The questionnaire was then completed *again* by all participants. To reduce the possibility of data contamination, trial sessions were held on the same day, one after another (meaning students would not encounter each other in between trial sessions). In addition, students receiving the digital intervention were unable to share the trial material with other students as it was pre-loaded onto the school’s laptops, which did not leave the classroom in which the trial was held. Initial data collection was conducted in January 2016, and six-month follow up assessments were conducted in July 2016, when the same questionnaire was again filled out by all participants.

### Experimental interventions

We trialled two different interventions; with Group A we trialled an interactive software package called *SimFection*, which uses computer simulations to illustrate concepts such as herd immunity, infectivity, mortality rates, the effect of migration, and ring vaccination (which are all covered in the current GCSE and A-Level Biology curricula). Diseases covered by SimFection include mumps, influenza, mumps and smallpox. SimFection is based on the SimZombie package [35], which has been successfully used by us for teaching and public engagement. This approach is based on the “health games” model [36, 37], which uses software [38], board games [39, 40] or other activities [41] to develop understanding of health-related issues.

The full package (including both Powerpoint presentations and the software) is freely available at http://www.simfection.org.uk [42]. With Group B we delivered a “traditional” Powerpoint-based presentation on infectious diseases (S1 Presentation).

For Group A, we used the *measles* simulation to illustrate the impact of different levels of vaccination coverage. At a low level of vaccination coverage, outbreaks occur and spread quickly through the population, and some agents in the simulation die, demonstrating the risk of infectious diseases to non-immunised people. When the vaccination coverage is set to a high value (above 95%), outbreaks are prevented, demonstrating to the user the effectiveness of vaccination at preventing the spread of infectious disease. Participants worked individually on the task, which was to find the minimum level of immunisation coverage needed to prevent a measles epidemic. This required the participants to use either trial-and-error or their previous knowledge of vaccination in order to establish that 95% is the minimum coverage level needed.

### Outcome measures

The questionnaire delivered before and after the interventions, and after a six month period, comprised two sections: an *attitudinal survey* (8 questions, using a Likert scale), and questions on *information needs and personal choice* (6 questions). The engagement survey, completed only after each intervention, comprised 6 questions.

The attitudinal score for each participant was generated from their responses to the first eight questions, which were scored as shown in Table 2. Each point on the scale was allocated a value in the range 1-5, and responses for each participant were summed to give an overall score. Higher scores signify a more *positive* attitude towards vaccination and the importance of protecting against infectious diseases, while low scores indicate mistrust of vaccination and/or negative views towards the need to protect populations. The maximum possible score was 40 (corresponding to someone having the most positive view of vaccination), and the lowest (most negative) possible score was 8 (assuming all questions were answered).

**Table 2:** Scoring system for attitudinal survey.

	Strongly Disagree	Disagree	Neutral	Agree	Strongly Agree
Vaccination can have serious side effects like causing disabilities in otherwise healthy children	5	4	3	2	1
The government would not let people get vaccinated if it was not safe	1	2	3	4	5
I would trust my doctor’s advice on vaccination	1	2	3	4	5
Vaccines contain unsafe ingredients	5	4	3	2	1
Diseases like measles are dangerous	1	2	3	4	5
It is important to get vaccinated to prevent the spread of infectious diseases throughout my community	1	2	3	4	5
Someone who isn’t vaccinated is likely to catch the infectious disease	1	2	3	4	5
People that don’t vaccinate themselves or their children put others at risk	1	2	3	4	5

The questions concerning information needs and personal choice (all scored on a Likert scale) are shown in Table 3. Engagement with each intervention was measured by the questions shown in Table 4 (also scored on a Likert scale).

**Table 3:** Scoring system for information needs and personal choice survey.

	Strongly Disagree	Disagree	Neutral	Agree	Strongly Agree
More information about vaccinations should be given to me	1	2	3	4	5
I know all I need to know about vaccination and how it works	1	2	3	4	5
Children should have more say than their parents when it comes to vaccinations	1	2	3	4	5
Someone under 16 who is well- informed should be able to choose to be (or not to be) vaccinated without their parent’s consent	1	2	3	4	5
It is nobody else’s business if I am vaccinated	1	2	3	4	5

**Table 4:** Scoring system for engagement survey.

	Strongly Disagree	Disagree	Neutral	Agree	Strongly Agree
I found the session informative	1	2	3	4	5
The session was interesting	1	2	3	4	5
I thought that the session was fun	1	2	3	4	5
I learnt something new from the session	1	2	3	4	5
The session was a good way for me to learn about infectious diseases	1	2	3	4	5

## Results

The initial group sizes were as follows: Group A (digital intervention), n = 26; Group B (presentation intervention), n = 21; Group C (control), n = 16. Subsequent absences or failure to engage with the follow-up questionnaire meant that we analysed complete results for 19, 17 and 16 individuals for Groups A, B and C respectively). The full data set of attitudinal scores is available in S1 Dataset, and the information/personal choice dataset in S2 Dataset.

The attitudinal analysis was performed as follows: for each participant, we compared their baseline (initial) attitudinal survey score with their post-intervention score, and their post-intervention score with their six-month follow-up score (that is, two comparisons per participant). Our summarised results are shown in Table 5; note that we only record the *direction* of attitudinal shift (or no shift), as we are not interested in absolute values. This allows us to sum over each comparison column in order to find the *number* of individuals who have changed attitudes.

**Table 5:** Shifts in attitude. For each participant within a group, we denote a positive attitudinal shift with “+”, a negative shift with “-”, and no shift with “0” (“x” denotes the fact that no response was recorded.

Digital Group (A)				PowerPoint Group (B)				Control Group (C)			
ID	Baseline	After Intervention	Follow Up	ID	Baseline	After Intervention	Follow Up	ID	Baseline	After Lesson	Follow Up
1	35	-	0	1	32	+	+	1	36	-	0
2	x	x	x	2	35	-	-	2	33	-	+
3	33	0	-	3	28	+	-	3	30	0	+
4	30	+	-	4	29	+	+	4	32	+	-
5	31	0	+	5	30	+	+	5	30	+	+
6	30	-	+	6	35	+	0	6	27	+	-
7	36	-	-	7	36	-	+	7	31	-	+
8	31	+	-	8	33	+	-	8	27	+	+
9	x	x	x	9	29	+	+	9	36	-	+
10	x	x	x	10	39	-	0	10	28	-	+
11	32	+	-	11	x	x	x	11	29	+	+
12	30	+	0	12	35	+	-	12	34	-	+
13	30	+	0	13	30	+	-	13	32	+	0
14	x	x	x	14	x	x	x	14	34	-	+
15	31	+	-	15	30	+	0	15	34	-	-
16	34	0	x	16	x	x	x	16	30	-	+
17	32	+	-	17	33	+	+				
18	32	-	+	18	35	-	-				
19	28	+	-	19	x	x	x				
20	x	x	x	20	32	-	+				
21	29	+	+	21	x	x	x				
22	30	+	-								
23	x	x	x								
24	x	x	x								
25	30	+	-								
26	x	x	x								

Chi-squared analysis revealed no statistically significant difference between the three groups after the intervention was delivered (p-0.115, df = 4). In addition, there was no statistically significant difference between the groups after the 6-month follow-up (p = 0.116, df = 4).

**Fig 1:**
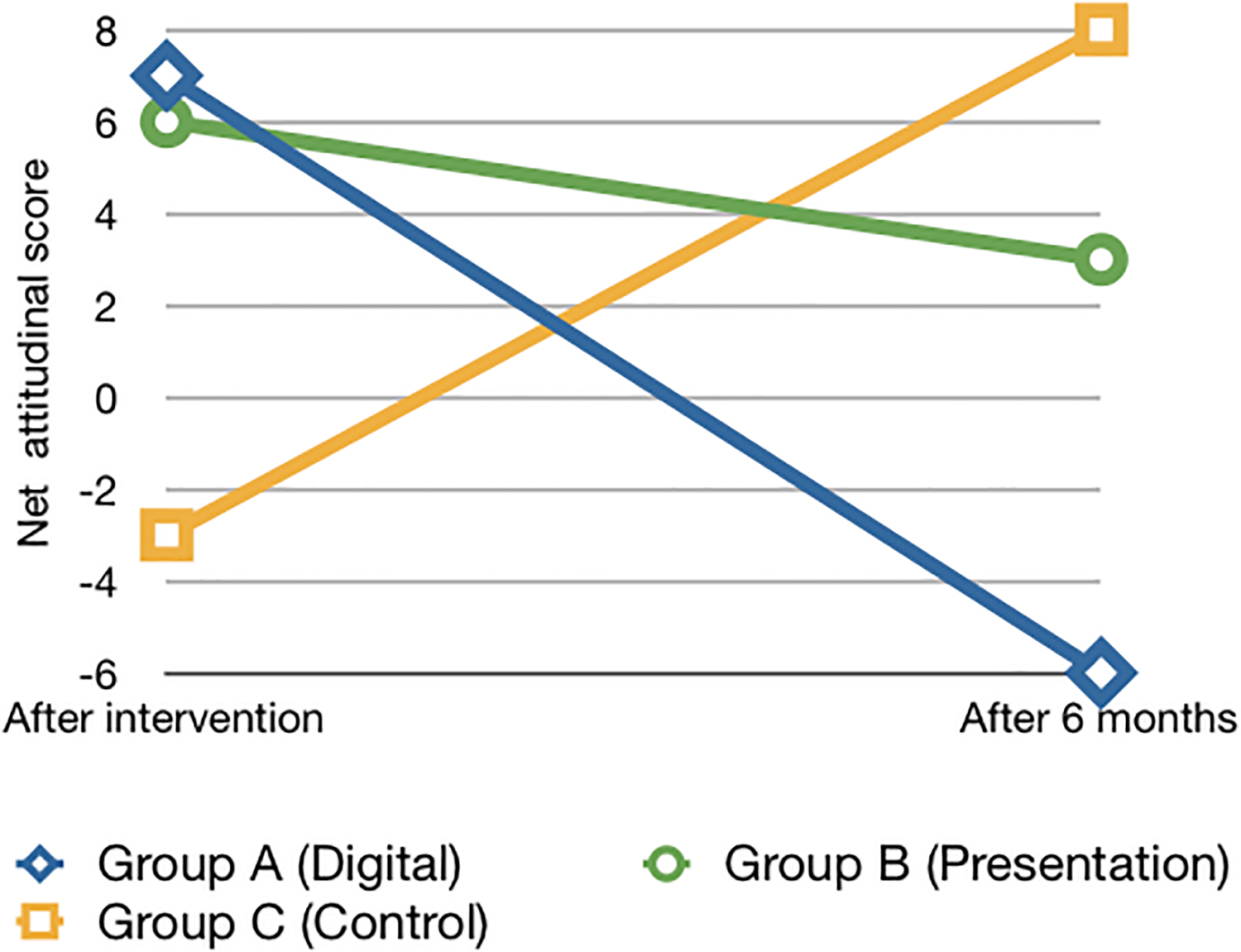
Net attitudinal shift.

In Fig 1 we show the net attitudinal shift per group over time (from after the intervention to the 6-month follow-up). For each group at each time point, we calculate the net attitudinal score by subtracting the number of negative shifts from the number of positive shifts. We see that both intervention groups have actually shifted to a *less* enthusiastic attitude towards vaccination, while the control group has moved to a more sympathetic position. We discuss the implications of this finding in the next Section.

We now consider the responses given to the questions concerning information needs and personal choice. Using Kruskal-Wallis analysis, we saw no statistical difference in responses across the trial groups to Q1, Q3, Q4, Q5 or Q6. We *did*, however, see a statistically significant difference between the three groups for Q2: “I know all I need to know about vaccination and how it works” (p = 0.004, df-8). Post-hoc analysis showed a significant difference between the simulation intervention group and the control group after six months (p = 0.044, df = 8), with fewer participants in the digital group agreeing with the statement after six months.

In terms of *engagement* with the interventions, using Mann-Whitney analysis we observed no statistically significant difference in responses to questions on this subject across the intervention groups, apart from Q1: “I found the session informative”, where more participants from the digital group agreed with the statement than in the presentation group (p = 0.04, df = 2). Qualitative written feedback received from participants focussed on a desire for *more information* about vaccination and its possible side-effects, and a need to use a wider range of example diseases. Several participants in Group A expressed a desire for more interactivity in the software. Although this feedback illuminates the design of the sessions, it does not fundamentally affect the findings with regard to attitudes.

## Discussion

Several initiatives have attempted to improve public attitudes towards vaccination. These have previously focused on adults (e.g., [43–45]), but a recent meta-analysis of previous vaccination interventions aimed at adults found that (a) they tend to have limited success, and (b) that they can, in some cases, actually *decrease* intent to vaccinate [16]. For this reason, and because of the gap in the literature covering young people, our research sought to assess the effectiveness of an educational intervention on attitudes in teenagers towards vaccination. This was deemed an appropriate age group to target, because it would reach a generally pre-parenthood group, members of which have expressed interest in receiving more information about vaccination [46].

When considering the format the intervention should take, the literature provided numerous examples of successful digital-based interventions for health. Notable examples include the “Re-mission” game, a digital health intervention, which has been shown to improve adherence to medical treatments and knowledge and understanding of cancer in young adults and adolescents with cancer [47], and a game (“PR:EPARe”) to be used in the classroom for Relationship and Sex Education [48].

Interviews were conducted with fourteen teenagers from the local area in order to explore teenagers’ attitudes towards vaccination. This provided a wealth of qualitative data that provided general themes that were significant to teenagers’ attitudes towards vaccination. These themes were: effectiveness of vaccination, safety of vaccination, risk of infectious disease, trust of healthcare professionals, information needs and personal choice. These were used to develop a series of statements about vaccination, which were refined into an eight-statement attitudinal survey. A focus group was used to test suitability for use with the target group.

We performed a full trial with three groups; one control, one receiving a digital resource-based intervention, and one receiving a traditional presentation-based intervention. We observed no significant differences between the three groups immediately after intervention, or after six months. For this reason, the main conclusion of our research is that educational interventions focussed on vaccination *do not* have a significant effect on the attitudes of young people. This conclusion is consistent with several recent studies; Nyhan, *et al.* showed that vaccination interventions aimed at *adults* have limited effectiveness [16], Dube, *et al.* showed that no available interventions could usefully address vaccine-hesitancy [12], and Fu, *et al.* could not find evidence to recommend any specific educational intervention to improve HPV vaccine acceptance [49]. However, a commentary on this latter paper [50] highlights the study of Marek, *et al.* [51], which demonstrated a positive impact on attitudes and intentions concerning HPV vaccination in young Hungarian adolescents. Although the the transferability of HPV-specific findings to a more general domain of vaccination remains an open question, it does suggest that, in certain contexts, educational interventions *can* have a positive effect on attitudes and behaviour.

Pre-intervention attitudinal scores were generally positive (simulation-based intervention group: 31.4/40; presentation-based intervention group: 32.5/40; Control group: 31.5/40), suggesting that this group was already well-disposed towards vaccination. This may account for the fact that there was no significant difference in attitudes after receiving the intervention.

Interestingly, there was no statistically significant difference between the digital resource group and the presentation-based intervention group in terms of engagement. This has significance for the current debate about the value of so-called “games for health” [15, 52]. The results from both test groups suggested that, in this particular study, the format of the intervention *did not* affect engagement levels.

The main limitation of this study was the sample size. For this reason, it is entirely possible that the study is underpowered and that some difference may have been observed between the trial groups had a larger sample size been used. All participants included in the trial were GCSE Biology students from the same school, with a focus on science education, so different results may have been obtained from a less engaged group of students. In addition, several ethnic and religious groups were under-represented; full trials conducted with a wider range of demographics would provide a clearer picture of the impact (if any) of different demographics. Additionally, this study bases its results on a one-time intervention, and it is possible that repeated interventions or a longer-term study would be more effective.

Some questions have emerged from this work:

1. Would vaccination interventions have a more significant effect on participants with more negative initial attitudes towards vaccination?
2. What, if any, effects do ethnicity and religious background have on the effectiveness of educational vaccination interventions?
3. If teenagers’ attitudes towards vaccination are generally positive, but vaccination uptake is lower than the recommended level set by the World Health Organisation, what other factors are negatively influencing attitudes towards vaccination between adolescence and parenthood?

In terms of the wider area of research, this project has demonstrated the difficulty of changing attitudes when using short-timescale interventions. This might suggest that more in-depth and/or longer-term interventions are needed to change complex attitudes such as attitudes towards vaccination.

## Supporting information

S1 Text Initial scoping interview schedule. (DOCX)

S1 Questionnaire Vaccination questionnaire. (DOCX)

S1 Worksheet Worksheet used with intervention groups. (DOCX)

S1 Presentation PowerPoint presentation used with Group B. (PPTX)

S1 Dataset Attitudinal scores dataset. (DOCX)

S2 Dataset Information needs/personal choice dataset. (DOCX)
