# Supplementary material for: Impact of educational interventions on adolescent attitudes and knowledge regarding vaccination: A pilot study
Source: PLoS One. 2018 Jan 19;13(1):e0190984. doi: 10.1371/journal.pone.0190984 (PMC5774691; doi:10.1371/journal.pone.0190984)
Supplement: S1 Text — (DOCX) [file pone.0190984.s001.docx]

S1: Initial Scoping Interview Schedule

Introduction

Hello, thank you for agreeing to be interviewed. Today I will ask you some questions about your views surrounding vaccination. If there is any question you would prefer not to answer, that is absolutely fine and you can withdraw from the interview at any time. The interview should take less than half an hour and will be tape recorded with your permission.

Background questions

First I would like to start with some background questions about you.

1. How old are you?
2. How would you describe your ethnicity?
3. What is your religious background?
4. Are you a student?

If yes: What are you currently studying?

If no: Are you currently in employment?

Main body of interview

1. Do you know what vaccination is? (Immunisation; Jabs)

If yes: Please can you describe how vaccination works?

Where does your knowledge of vaccination come from? Did you learn about it in school?

If no: Define vaccination to interviewee (“is when a weakened virus or bacterium, a part of a virus or bacterium, or a weakened product of a bacterium is deliberately administered to you (normally by injection) so that your immune system can prepare to fight a future infection”)

1. Can I ask if you remember ever being vaccinated? Or know that you have been? (When you were a child or a teenager?)

If yes: How old were you?

What happened?

Do you remember anyone discussing vaccination with you? Who?

If no: Do you know why not?

1. Do you think vaccinations are safe? (Very safe? Not very safe?)
2. What do you think the risks of vaccination are?
   Why do you think that?
3. Do you worry about the spread of infectious diseases? (Such as measles, mumps, and influenza?)

Why/Why not?

1. Why do you think some people are against vaccination? (Some people are worried about the risks of vaccination or think that vaccines are not necessary)
2. What are your opinions on the reasons that people have for not vaccinating?
3. Do you think parents should have the right to choose not to vaccinate their children?
4. If you needed to make a decision about whether to personally vaccinate or not, where would you look for information? (For example, family, friends, internet, and news reports?)
5. How would you know if an information source is reliable?
6. Do you ever discuss heath issues such as vaccination?
   Who with?
7. Would your family’s opinions on vaccination influence your attitude towards vaccination?
   (For example, if your parents were for or against vaccination would that make you more or less likely to vaccinate?)
   Why/why not?
8. Would your friends’ opinions on vaccination influence your attitude towards vaccination?
   (For example, if your friends were for or against vaccination would that make you more or less likely to vaccinate?)
   Why/why not?
9. Would your doctor’s opinions on vaccination influence your attitude towards vaccination?
   (For example, if your doctor was for or against vaccination would that make you more or less likely to vaccinate?)
   Why/why not?
10. If religious, do your religious views influence your views on vaccination?

Why/why not?

1. What do you think influences your views on vaccination the most? (For example media reports)
2. Do you have any ethical concerns about vaccination? (For example testing on animals, adults making decisions on behalf of their children?)
   Do these issues impact your views on vaccination?
3. Do you have any concerns about sterility or cleanliness of vaccination?
4. Is there anything else that worries you about vaccination? (For example pain? Fear of needles? Ingredients of the vaccine?)
5. Do you think there are any specific vaccines that you would not accept? (For example polio? Tetanus? Measles?)
   Why?
6. What do you think would make you feel more confident about vaccination? (For example, more information about its safety?)
7. If you decide to have children in the future, do you think you will vaccinate them?
   What factors would influence your decision?
