# Supplementary material for: Impact of educational interventions on adolescent attitudes and knowledge regarding vaccination: A pilot study
Source: PLoS One. 2018 Jan 19;13(1):e0190984. doi: 10.1371/journal.pone.0190984 (PMC5774691; doi:10.1371/journal.pone.0190984)
Supplement: S1 Questionnaire — (DOCX) [file pone.0190984.s002.docx]

S2: Vaccination Questionnaire

Background information

1. Age
2. Gender (Please tick one)
   Male Female Other Prefer not to say
3. Ethnicity (Please tick one)

*White*

1. Welsh/English/Scottish/Northern Irish/British 
2. Irish 
3. Gypsy or Irish Traveller 
4. Any other White background, please describe ……..

*Mixed/Multiple ethnic groups*

5. White and Black Caribbean 
6. White and Black African 
7. White and Asian 
8. Any other Mixed/Multiple ethnic background, please describe ………..

*Asian/Asian British*

9. Indian 
10. Pakistani 
11. Bangladeshi 
12. Chinese 
13. Any other Asian background, please describe ……….

*Black/African/Caribbean/Black British*

14. African 
15. Caribbean 
16. Any other Black/African/Caribbean background, please describe ………

*Other ethnic group*

17. Arab 
18. Any other ethnic group, please describe …….

1. Religious Background (Please tick one)
   1. Christian (including Church of England, Catholic, Protestant and all other Christian denominations)
   2. Buddhist
   3. Hindu
   4. Jewish
   5. Muslim
   6. Sikh
   7. No religion
   8. Prefer not to say

Knowledge of vaccination

Can you describe what is injected into you when you are vaccinated? ………………………………………………………………………………………………………………………………………………………………………………………………………………………………………………………………………………………………………………………..

Can you explain why a person won’t get ill if they have been vaccinated?

……………………………………………………………………………………………………………………………………………………………………………………………………………………………………………………………………………………………………………………

What do you think is a benefit of vaccinating a large percentage of people?

………………………………………………………………………………………………………………………………………………………………………………………………………………………………………………………………………………………………………………

Can you think of any medical reasons a person would not be able to be vaccinated?

…………………………………………………………………………………………………………………………………………………………………………………………………………………………………………………………………………………………………………………

Can you explain why ‘booster’ vaccinations are sometimes needed?

……………………………………………………………………………………………………………………………………………………………………………………………………………………………………………………………………………………………………………………

Why can’t a vaccine be made against some types of viruses like flu?

……………………………………………………………………………………………………………………………………………………………………………………………………………………………………………………………………………………………………………………

Can you think of any potential risks of vaccination?

………………………………………………………………………………………………………………………………………………………………………………………………………………………………………………………………………………………………………………………..

Attitude towards vaccination

Please tick one response per row

|  | Strongly Disagree | Disagree | Neither agree nor disagree | Agree | Strongly Agree |
| --- | --- | --- | --- | --- | --- |
| Vaccination can have serious side effects like causing disabilities in otherwise healthy people |  |  |  |  |  |
| The government would not let people get vaccinated if it was not safe |  |  |  |  |  |
| I would trust my doctor’s advice on vaccination |  |  |  |  |  |
| Vaccines contain unsafe ingredients |  |  |  |  |  |
| Diseases like measles are dangerous |  |  |  |  |  |
| It is important to get vaccinated to prevent the spread of infectious diseases through my community |  |  |  |  |  |
| Someone who isn’t vaccinated is likely to catch the infectious disease |  |  |  |  |  |
| People that don’t vaccinate themselves or their children put others at risk |  |  |  |  |  |
| More information about vaccinations should be given to me |  |  |  |  |  |
| I know all I need to know about vaccination and how it works |  |  |  |  |  |
| Children should have more say than their parents should when it comes to their own vaccinations |  |  |  |  |  |
| Someone under 16 who is well informed should be able to choose to be (or not be) vaccinated without their parent’s consent |  |  |  |  |  |
| Doctors, not parents or their children, should have the final say about if a child is vaccinated |  |  |  |  |  |
| It is nobody else’s business if I am vaccinated |  |  |  |  |  |
