# Supplementary material for: Impact of educational interventions on adolescent attitudes and knowledge regarding vaccination: A pilot study
Source: PLoS One. 2018 Jan 19;13(1):e0190984. doi: 10.1371/journal.pone.0190984 (PMC5774691; doi:10.1371/journal.pone.0190984)
Supplement: S1 Worksheet — (DOCX) [file pone.0190984.s003.docx]

S3: Vaccination worksheet

1. What is vaccination?

………………………………………………………………………………

………………………………………………………………………………

………………………………………………………………………………

1. What is the benefit of vaccinating?

………………………………………………………………………………

………………………………………………………………………………

………………………………………………………………………………

1. Why can’t some people be vaccinated?

………………………………………………………………………………

………………………………………………………………………………

………………………………………………………………………………

1. What is it called when vaccination coverage is high enough to prevent outbreaks?

………………………………………………………………………………

………………………………………………………………………………

………………………………………………………………………………

1. What percentage of vaccination coverage do we need to prevent outbreaks of measles?

………………………………………………………………………………

………………………………………………………………………………

………………………………………………………………………………

1. What happens if vaccination coverage is low?

………………………………………………………………………………

………………………………………………………………………………

………………………………………………………………………………
