# Supplementary material for: Impact of educational interventions on adolescent attitudes and knowledge regarding vaccination: A pilot study
Source: PLoS One. 2018 Jan 19;13(1):e0190984. doi: 10.1371/journal.pone.0190984 (PMC5774691; doi:10.1371/journal.pone.0190984)
Supplement: S1 Presentation — (PPTX) [file pone.0190984.s004.pptx]

## Slide 1
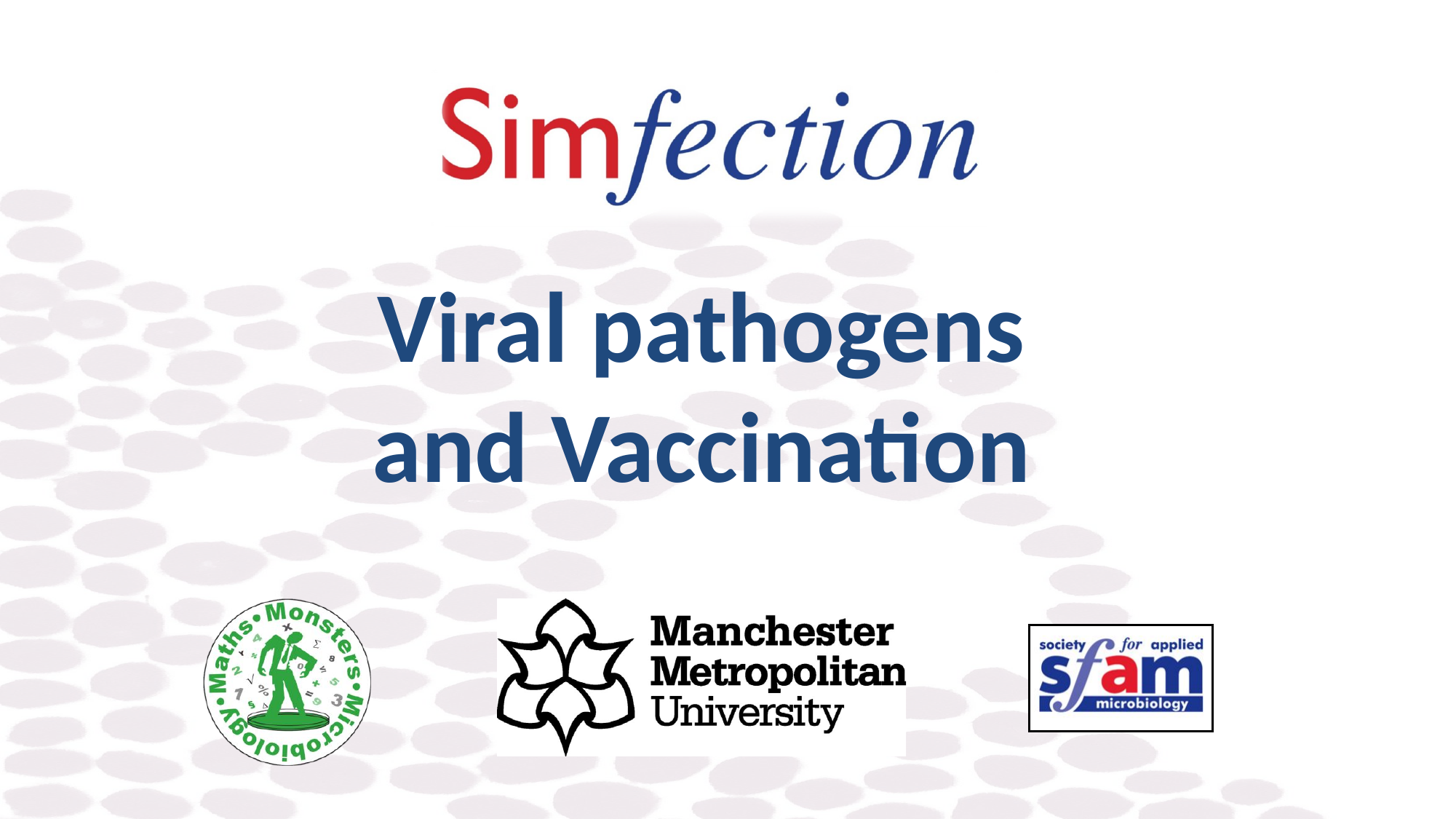

# Viral pathogens and Vaccination

## Slide 2
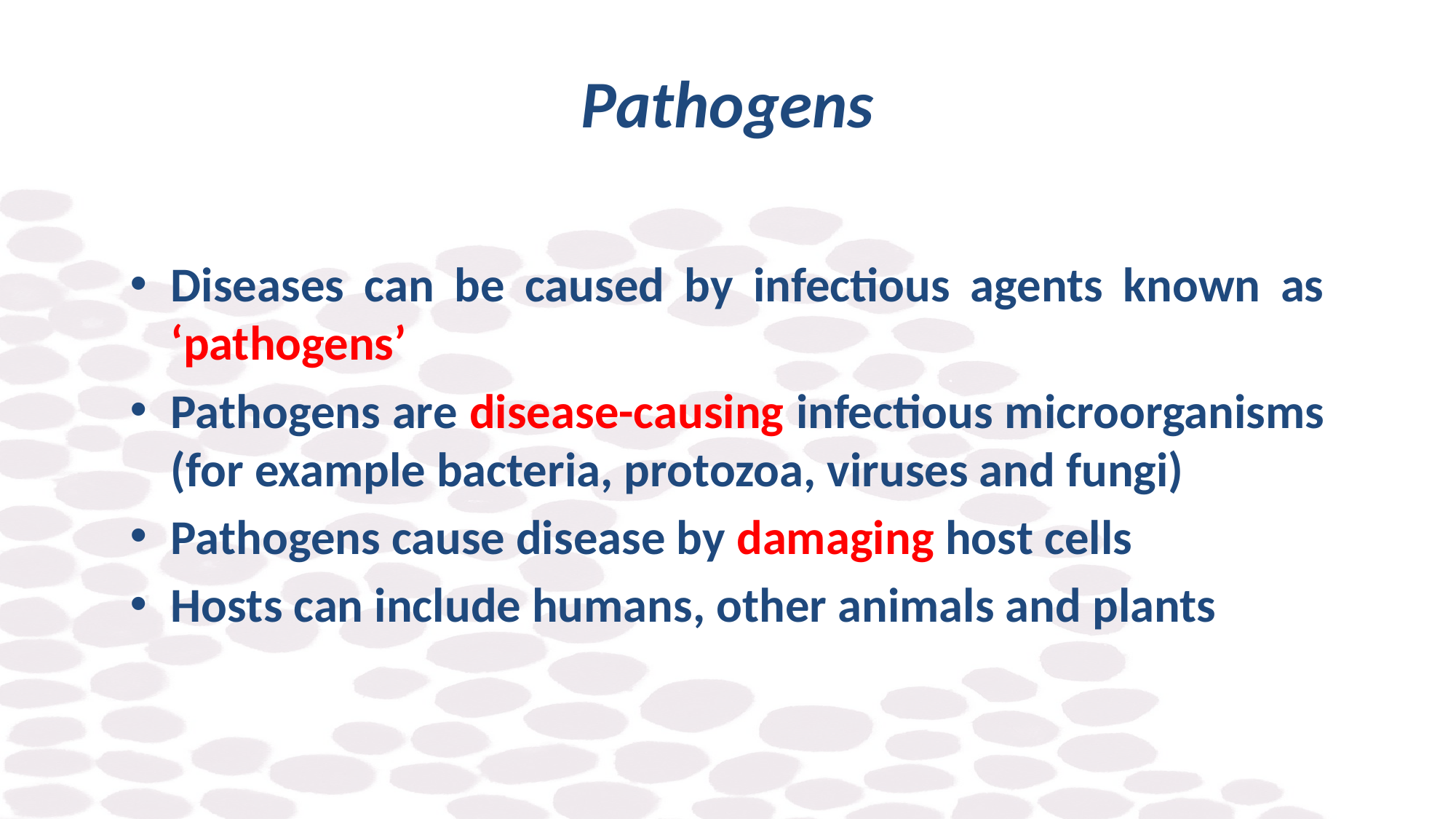

# Pathogens
Diseases can be caused by infectious agents known as ‘pathogens’
Pathogens are disease-causing infectious microorganisms (for example bacteria, protozoa, viruses and fungi)
Pathogens cause disease by damaging host cells
Hosts can include humans, other animals and plants

## Slide 3
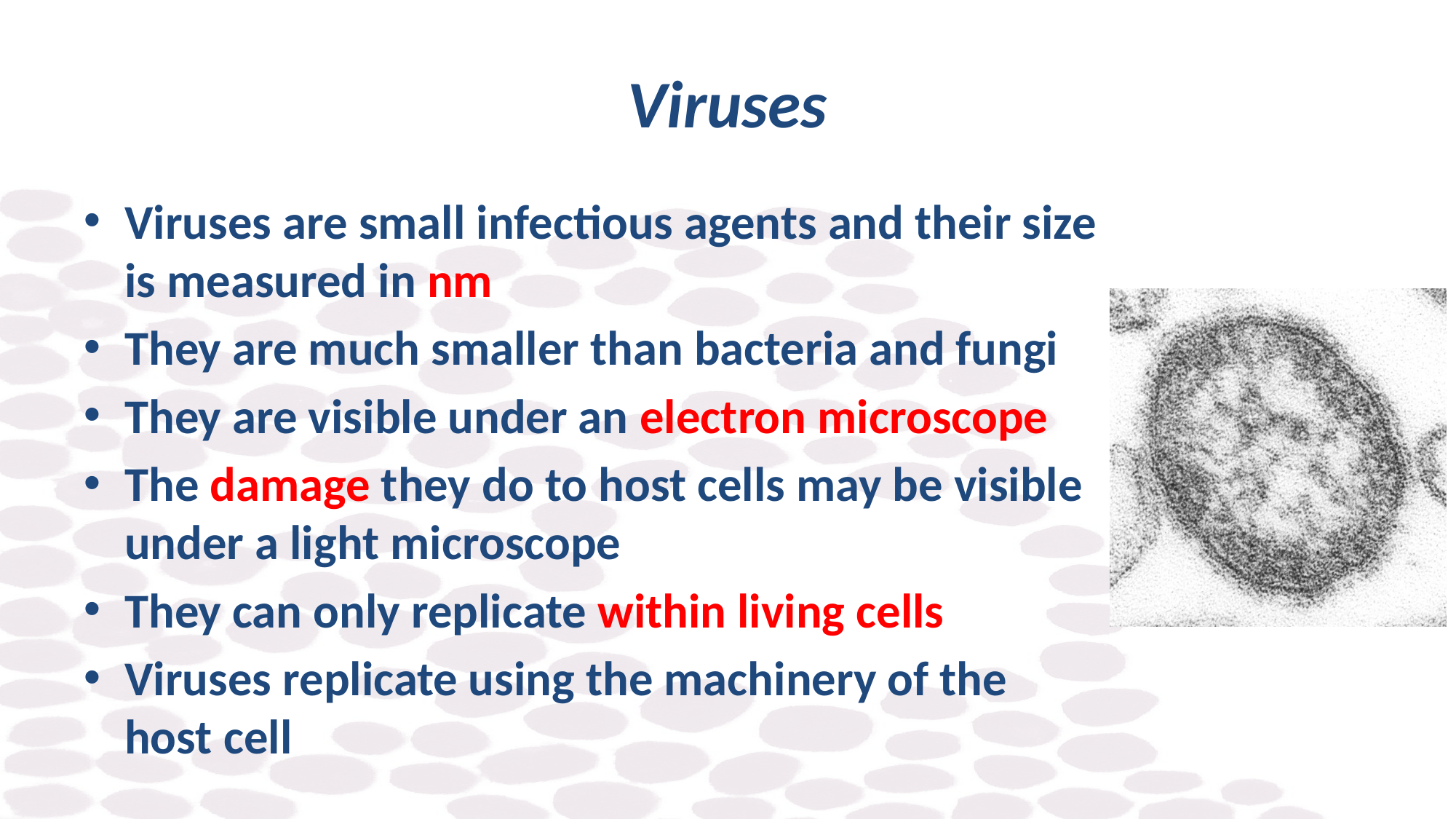

# Viruses
Viruses are small infectious agents and their size is measured in nm
They are much smaller than bacteria and fungi
They are visible under an electron microscope
The damage they do to host cells may be visible under a light microscope
They can only replicate within living cells
Viruses replicate using the machinery of the host cell

## Slide 4
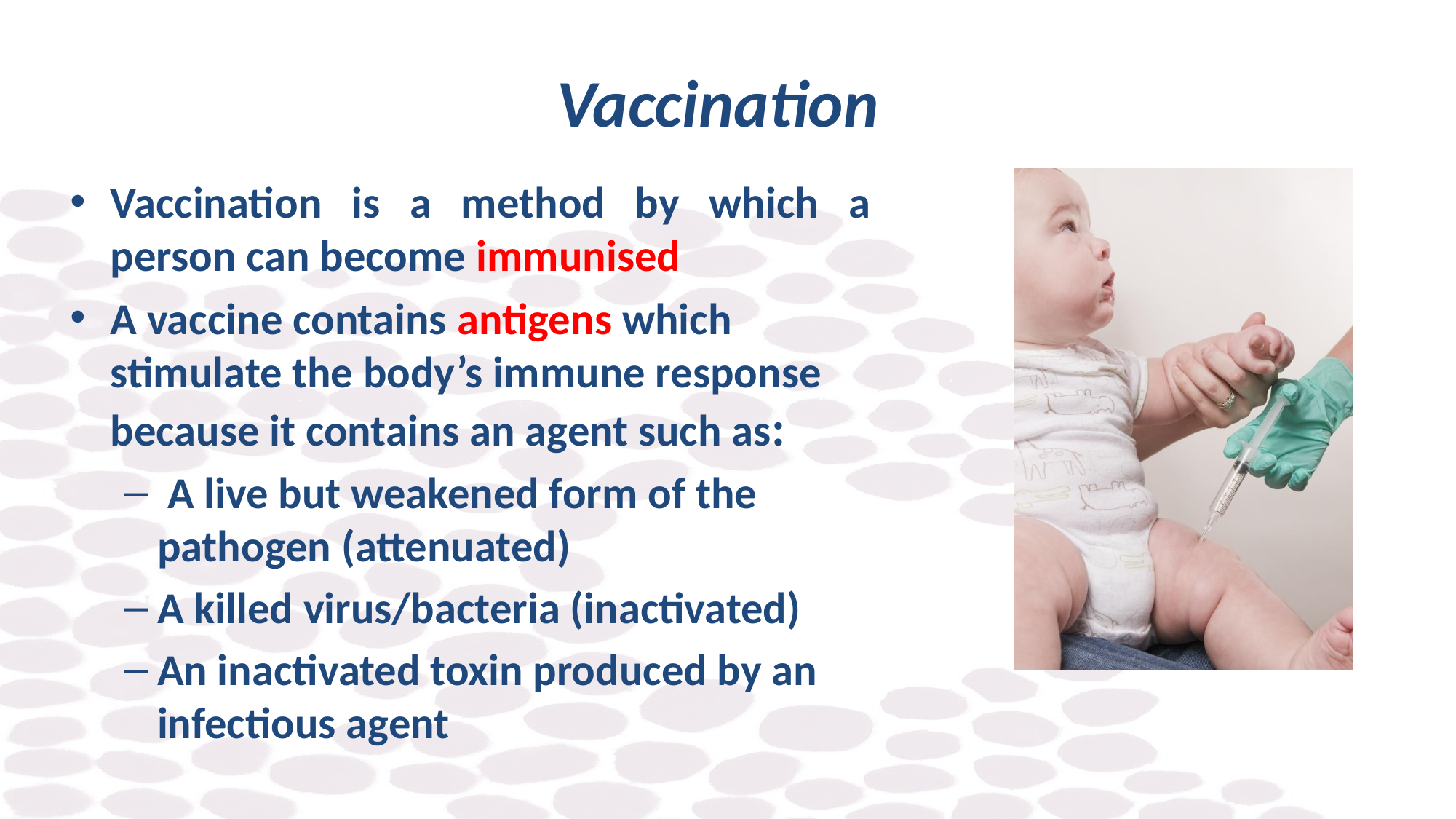

# Vaccination
Vaccination is a method by which a person can become immunised
A vaccine contains antigens which stimulate the body’s immune response because it contains an agent such as:
 A live but weakened form of the pathogen (attenuated)
A killed virus/bacteria (inactivated)
An inactivated toxin produced by an infectious agent

## Slide 5
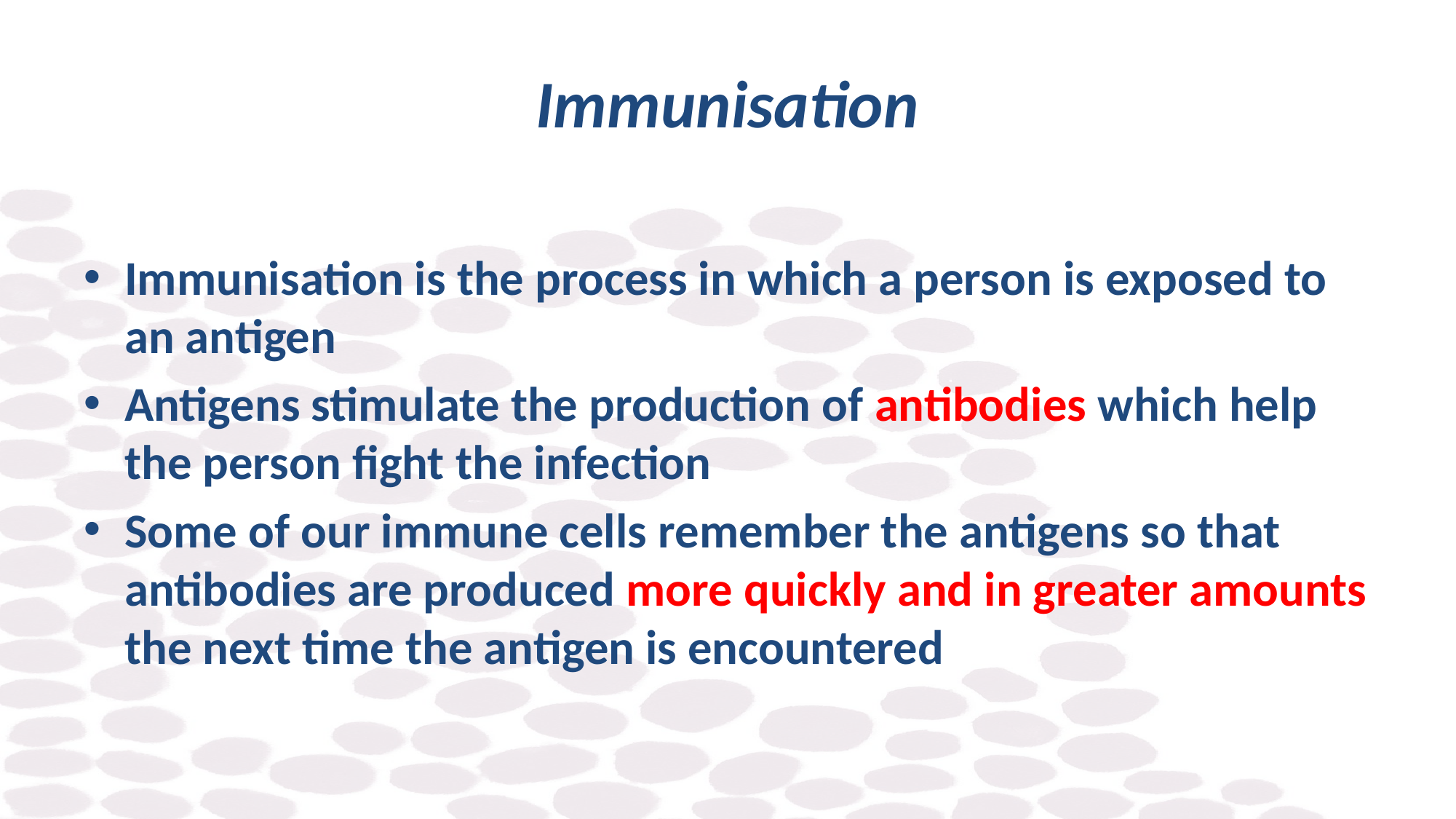

# Immunisation
Immunisation is the process in which a person is exposed to an antigen
Antigens stimulate the production of antibodies which help the person fight the infection
Some of our immune cells remember the antigens so that antibodies are produced more quickly and in greater amounts the next time the antigen is encountered

## Slide 6
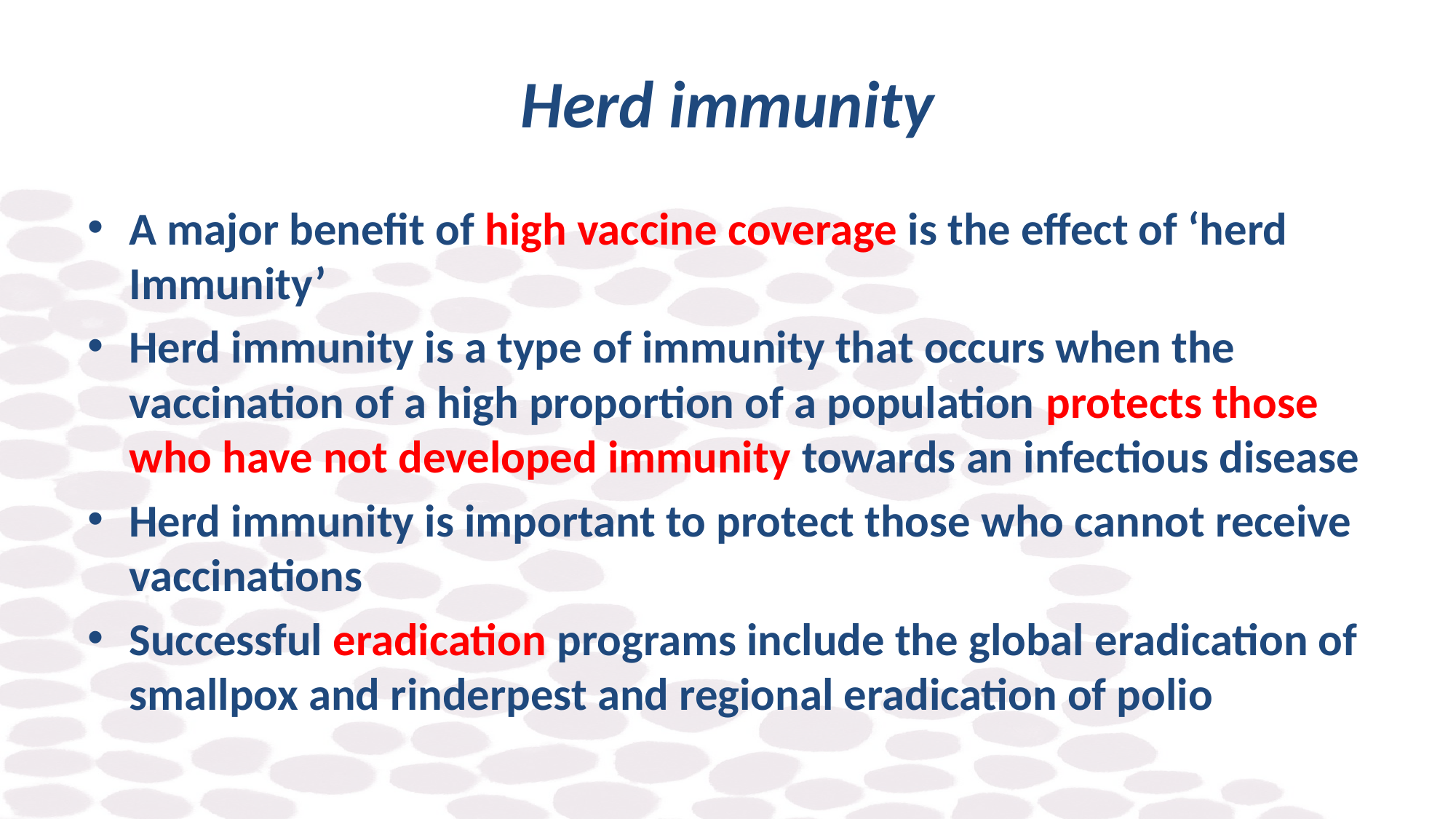

# Herd immunity
A major benefit of high vaccine coverage is the effect of ‘herd Immunity’
Herd immunity is a type of immunity that occurs when the vaccination of a high proportion of a population protects those who have not developed immunity towards an infectious disease
Herd immunity is important to protect those who cannot receive vaccinations
Successful eradication programs include the global eradication of smallpox and rinderpest and regional eradication of polio

## Slide 7
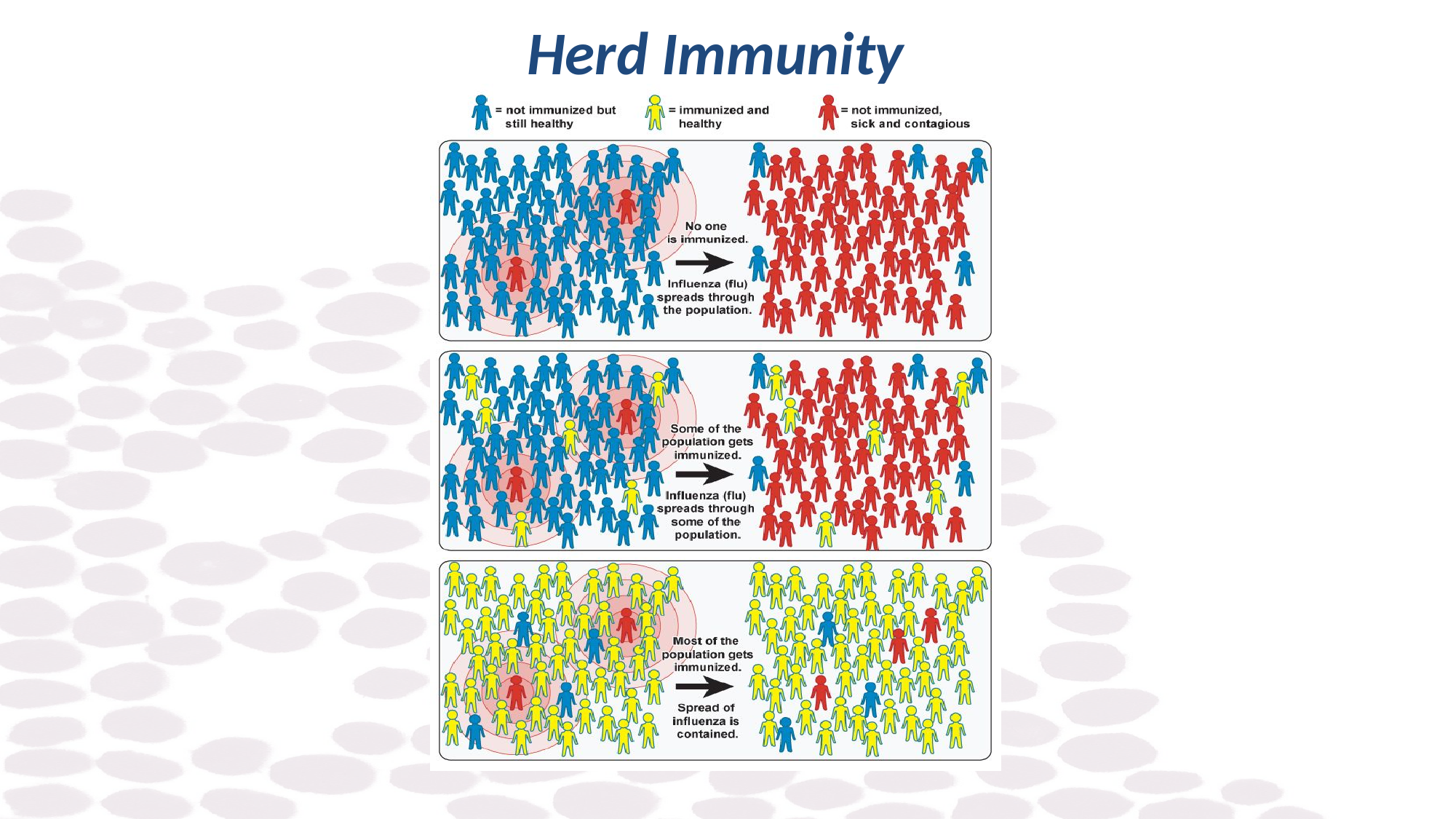

# Herd Immunity

## Slide 8
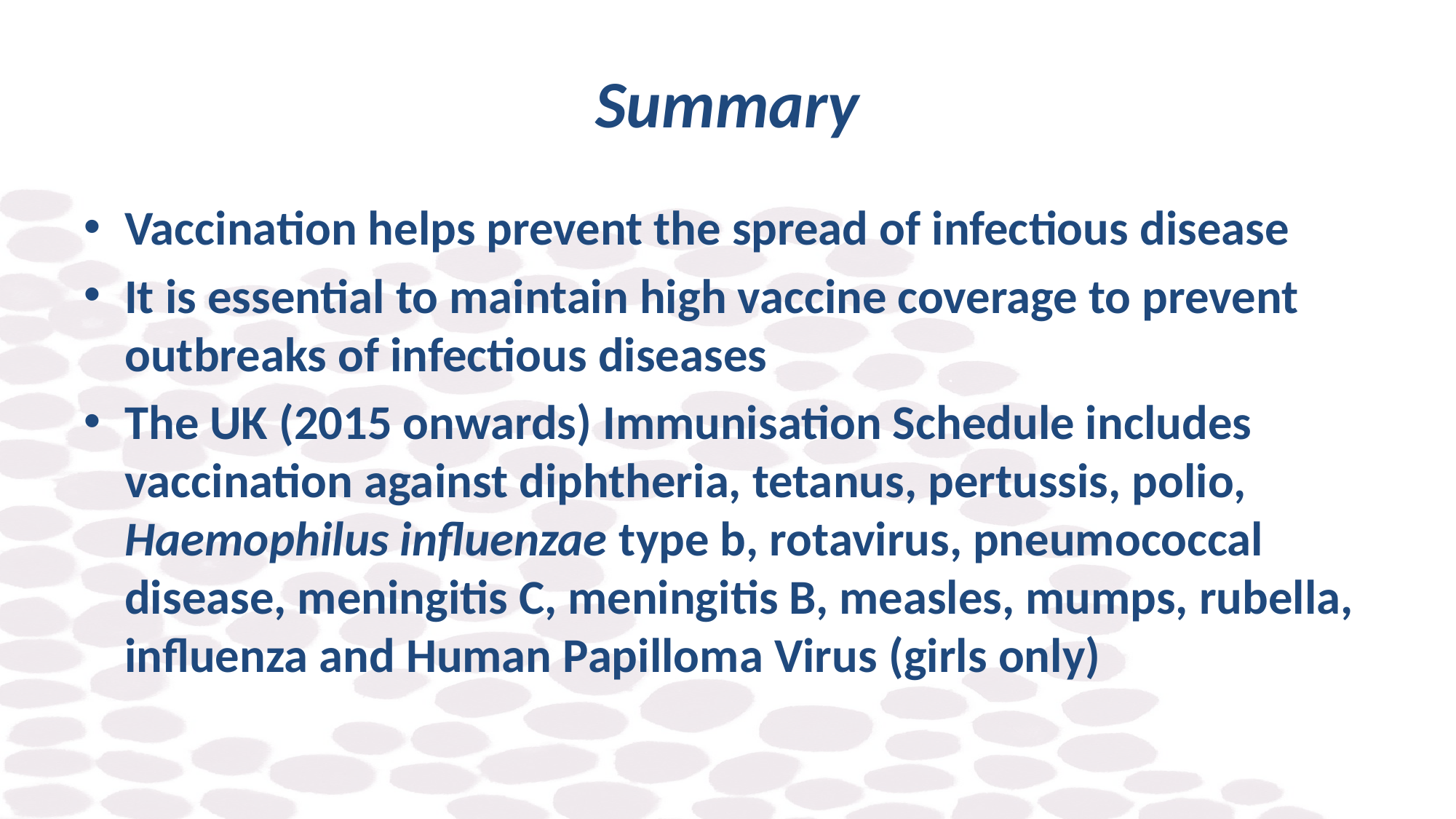

# Summary
Vaccination helps prevent the spread of infectious disease
It is essential to maintain high vaccine coverage to prevent outbreaks of infectious diseases
The UK (2015 onwards) Immunisation Schedule includes vaccination against diphtheria, tetanus, pertussis, polio, Haemophilus influenzae type b, rotavirus, pneumococcal disease, meningitis C, meningitis B, measles, mumps, rubella, influenza and Human Papilloma Virus (girls only)

## Slide 9
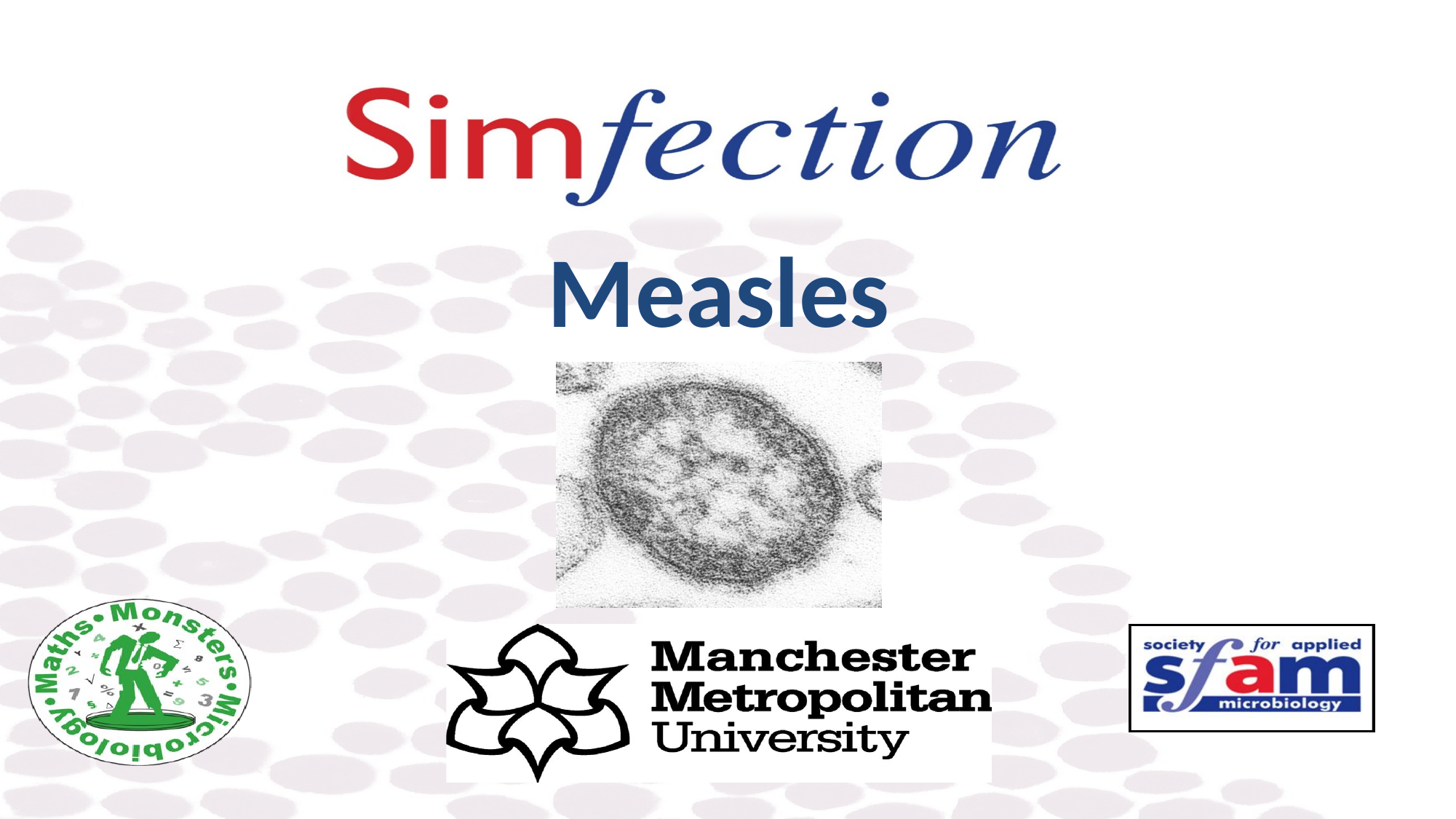

# Measles

## Slide 10
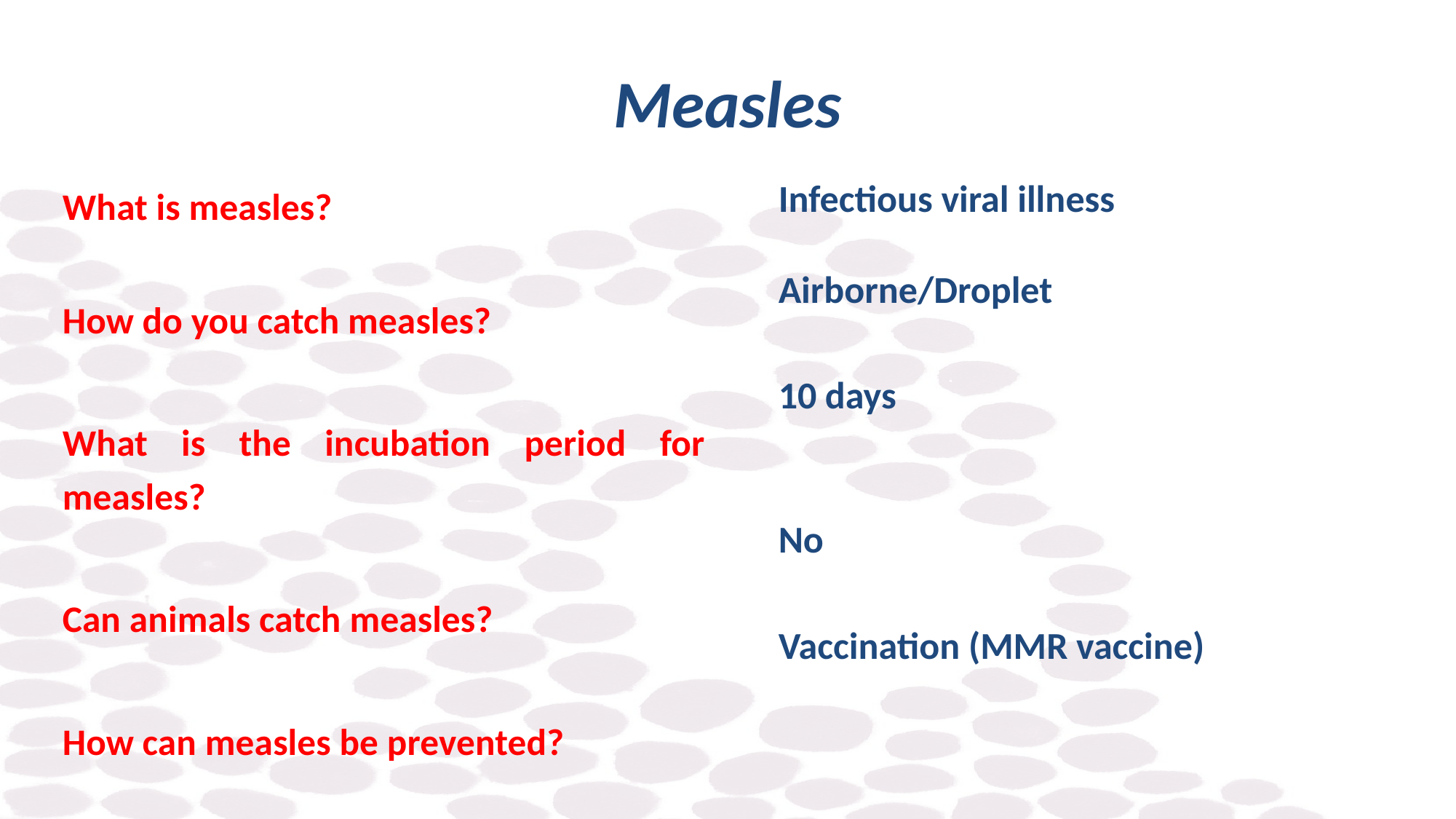

# Measles
What is measles?
How do you catch measles?
What is the incubation period for measles?
Can animals catch measles?
How can measles be prevented?
Infectious viral illness
Airborne/Droplet
10 days
No
Vaccination (MMR vaccine)

## Slide 11
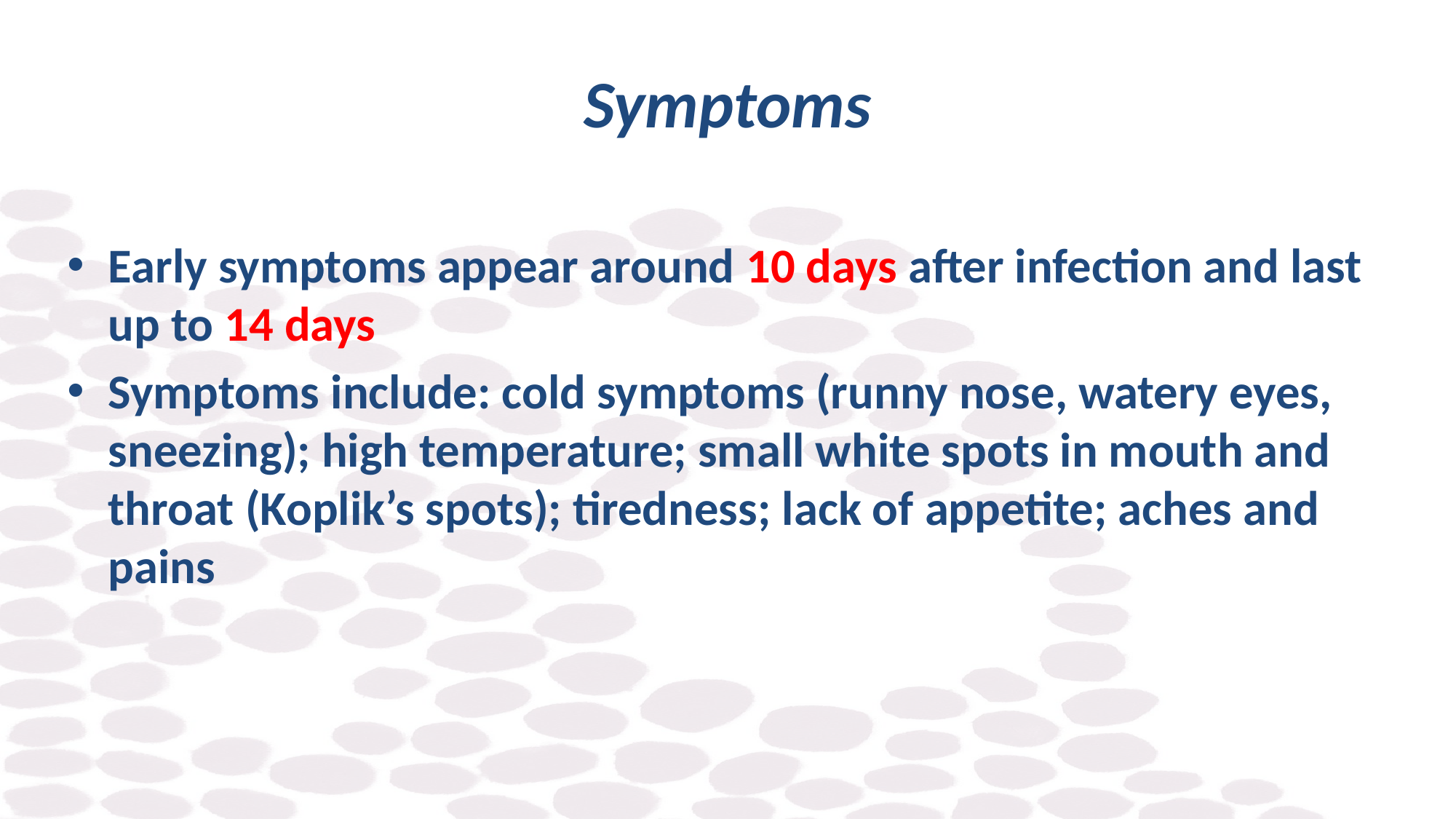

# Symptoms
Early symptoms appear around 10 days after infection and last up to 14 days
Symptoms include: cold symptoms (runny nose, watery eyes, sneezing); high temperature; small white spots in mouth and throat (Koplik’s spots); tiredness; lack of appetite; aches and pains

## Slide 12
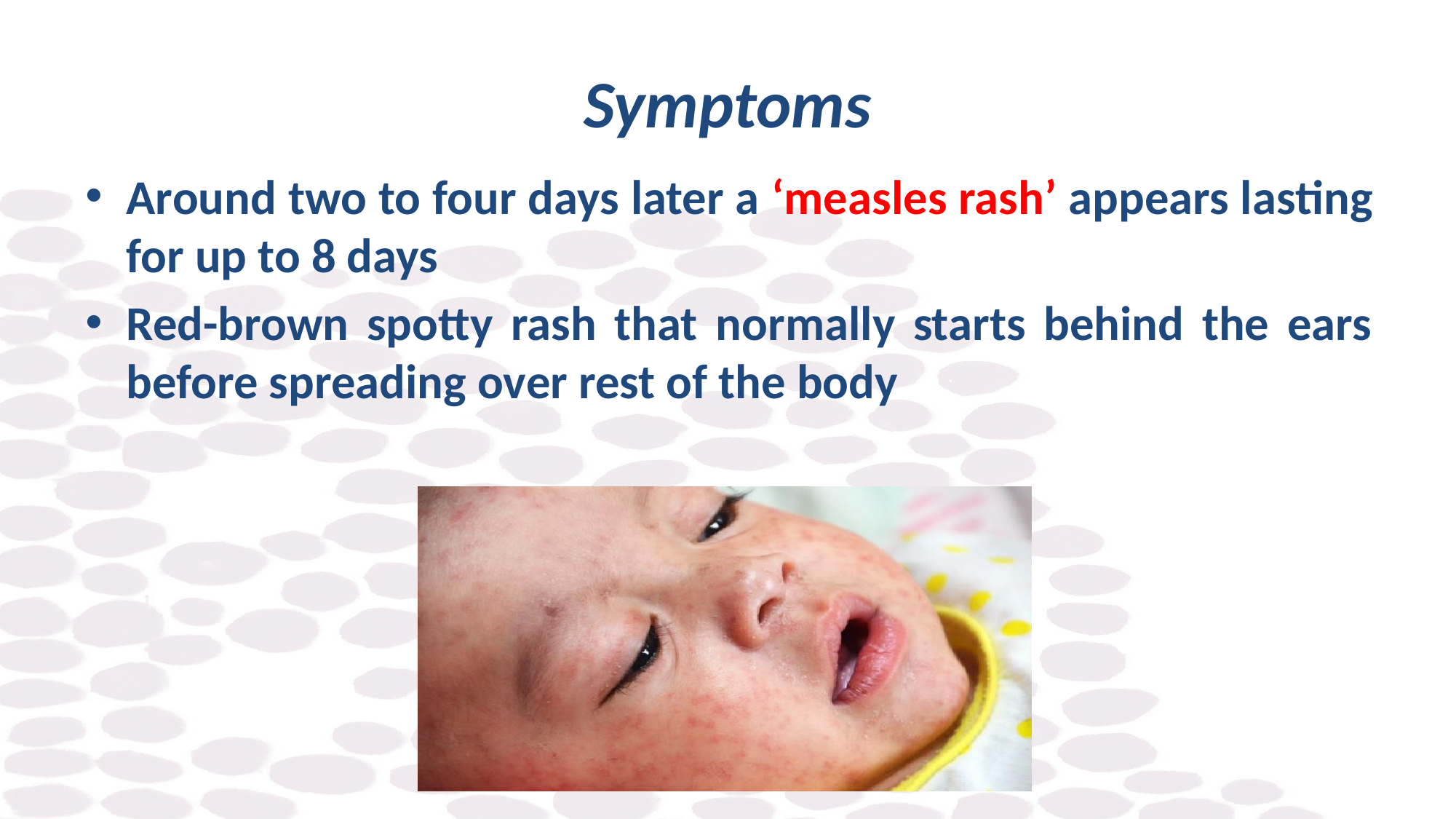

# Symptoms
Around two to four days later a ‘measles rash’ appears lasting for up to 8 days
Red-brown spotty rash that normally starts behind the ears before spreading over rest of the body

## Slide 13
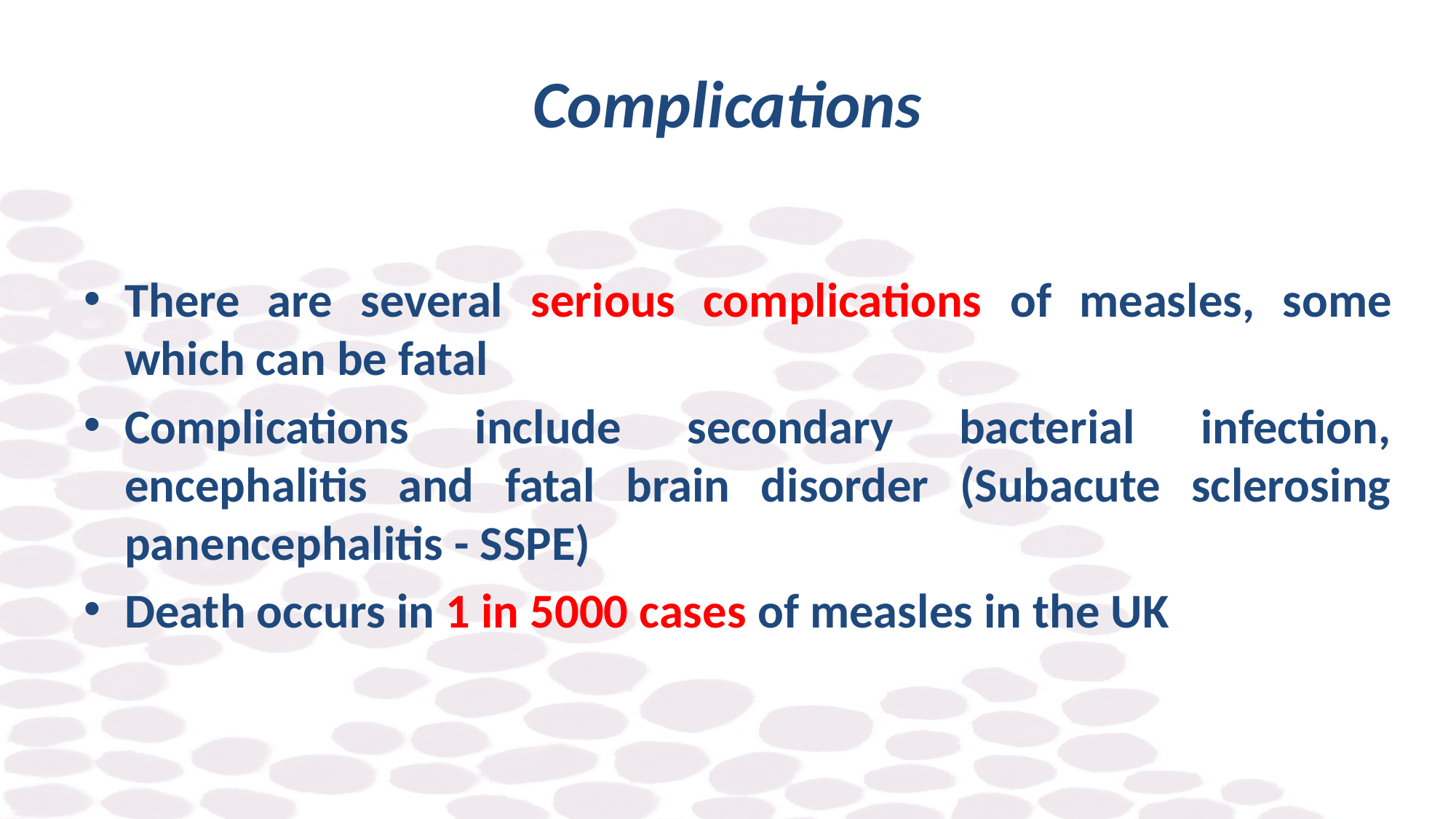

# Complications
There are several serious complications of measles, some which can be fatal
Complications include secondary bacterial infection, encephalitis and fatal brain disorder (Subacute sclerosing panencephalitis - SSPE)
Death occurs in 1 in 5000 cases of measles in the UK

## Slide 14
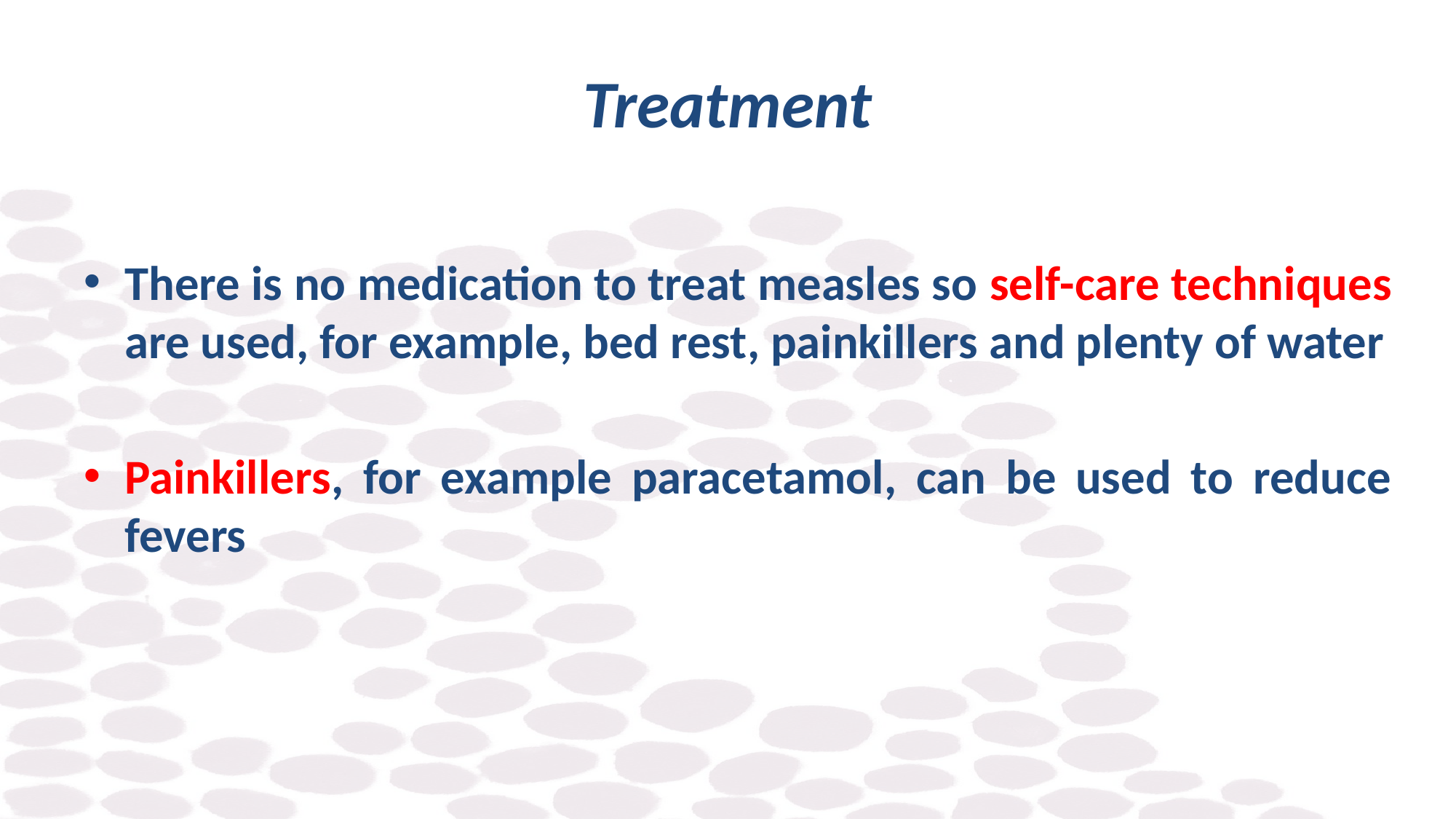

# Treatment
There is no medication to treat measles so self-care techniques are used, for example, bed rest, painkillers and plenty of water
Painkillers, for example paracetamol, can be used to reduce fevers

## Slide 15
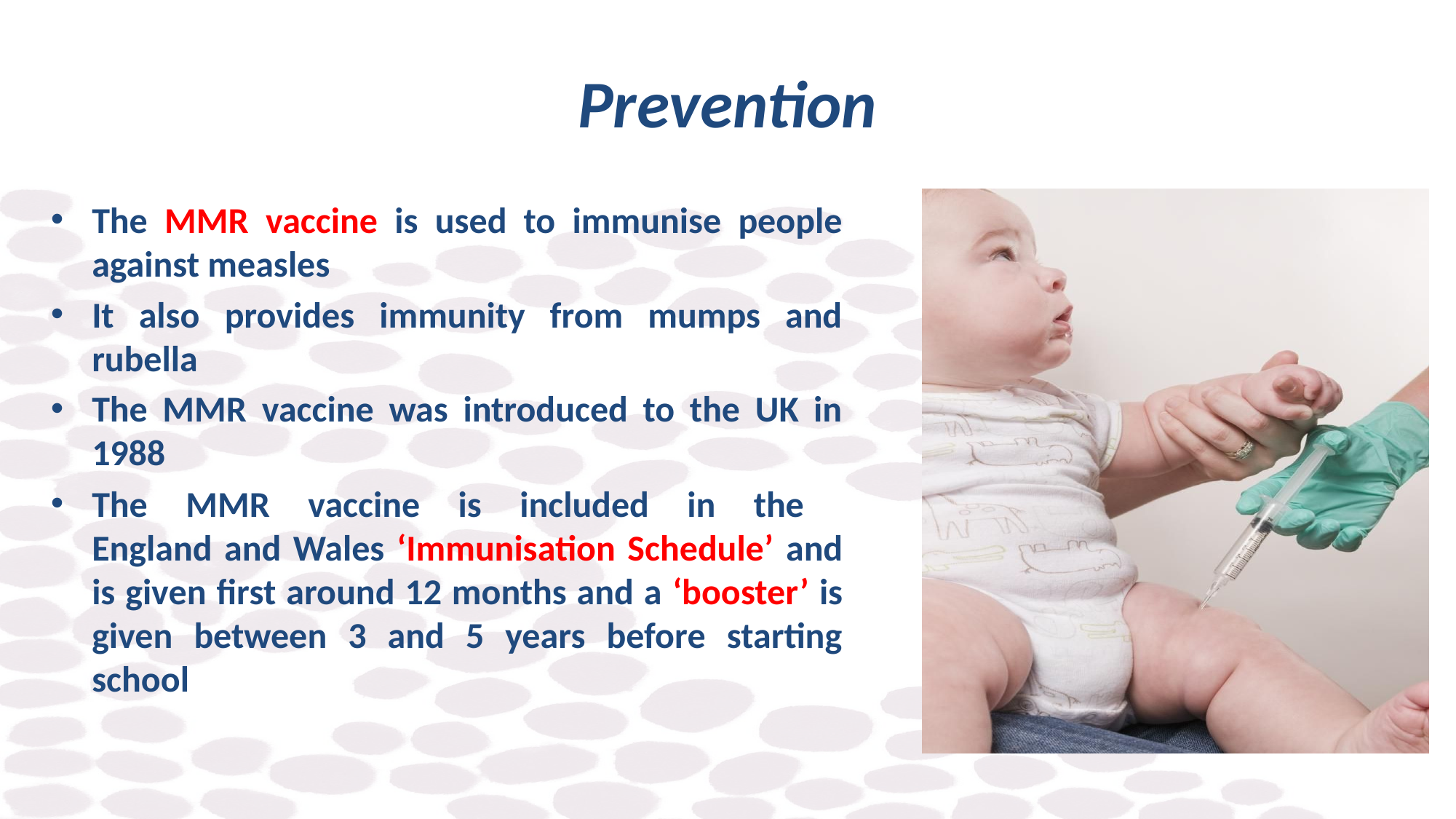

# Prevention
The MMR vaccine is used to immunise people against measles
It also provides immunity from mumps and rubella
The MMR vaccine was introduced to the UK in 1988
The MMR vaccine is included in the
England and Wales ‘Immunisation Schedule’ and is given first around 12 months and a ‘booster’ is given between 3 and 5 years before starting school

## Slide 16
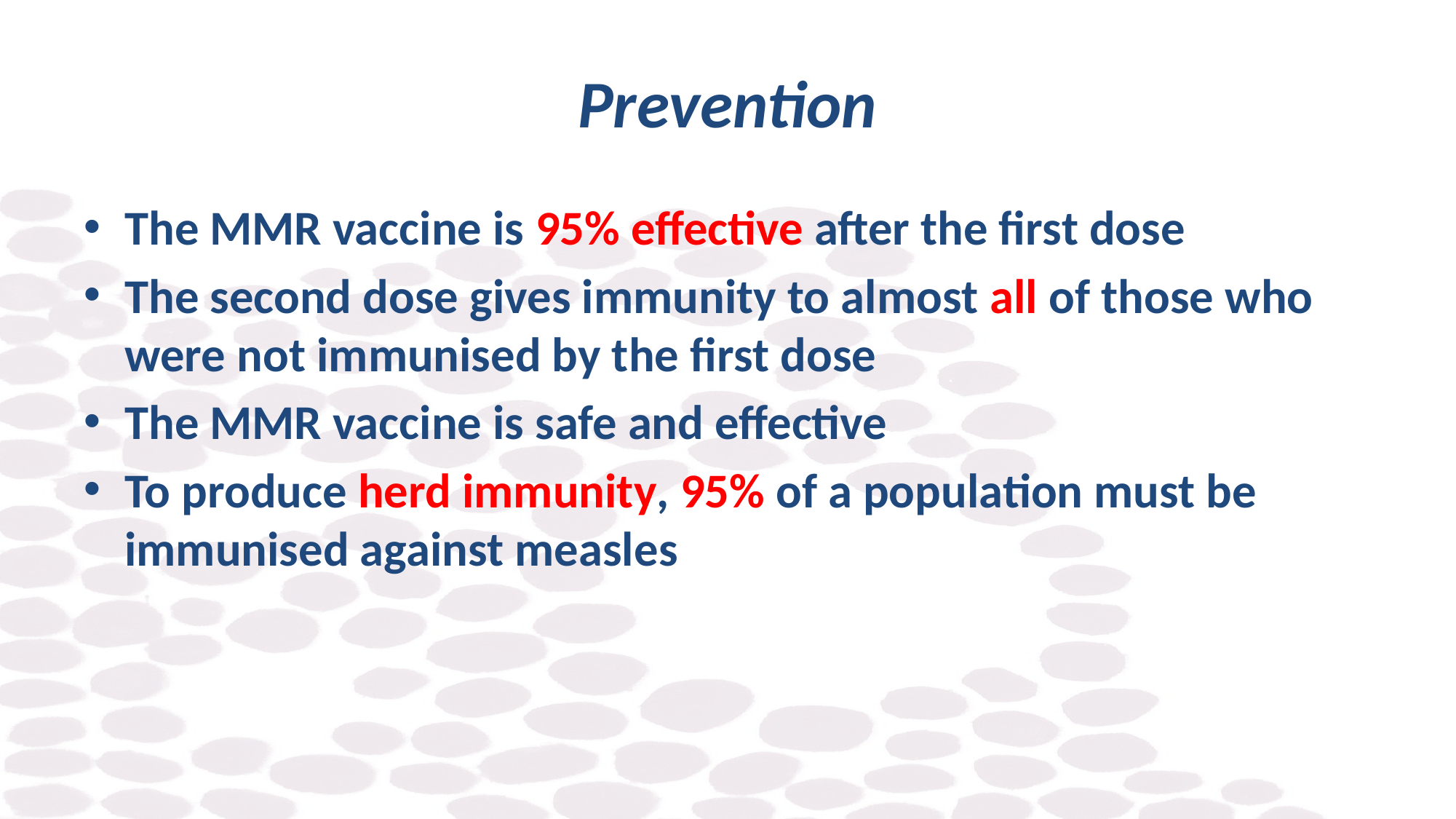

# Prevention
The MMR vaccine is 95% effective after the first dose
The second dose gives immunity to almost all of those who were not immunised by the first dose
The MMR vaccine is safe and effective
To produce herd immunity, 95% of a population must be immunised against measles

## Slide 17
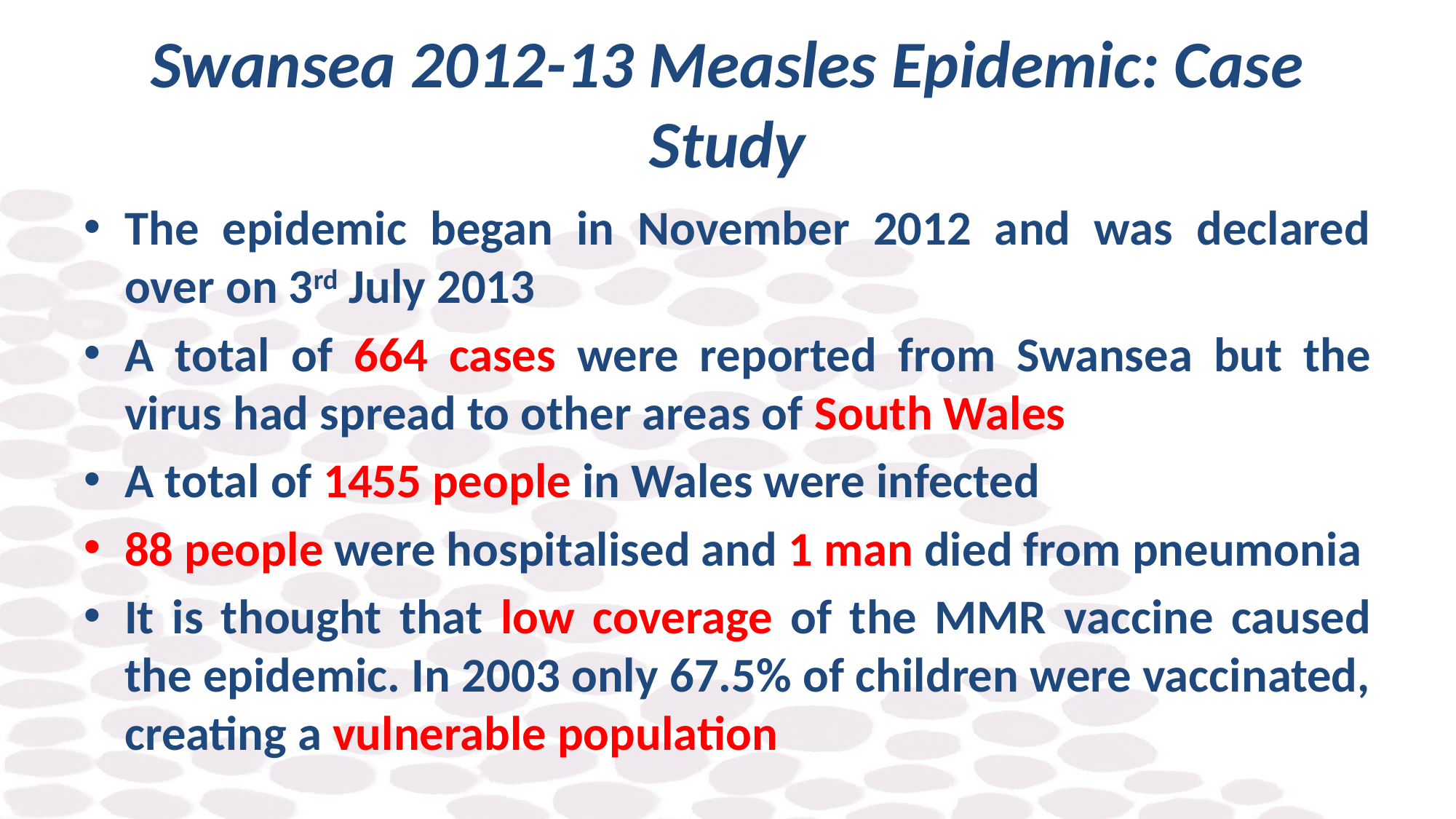

# Swansea 2012-13 Measles Epidemic: Case Study
The epidemic began in November 2012 and was declared over on 3rd July 2013
A total of 664 cases were reported from Swansea but the virus had spread to other areas of South Wales
A total of 1455 people in Wales were infected
88 people were hospitalised and 1 man died from pneumonia
It is thought that low coverage of the MMR vaccine caused the epidemic. In 2003 only 67.5% of children were vaccinated, creating a vulnerable population

## Slide 18
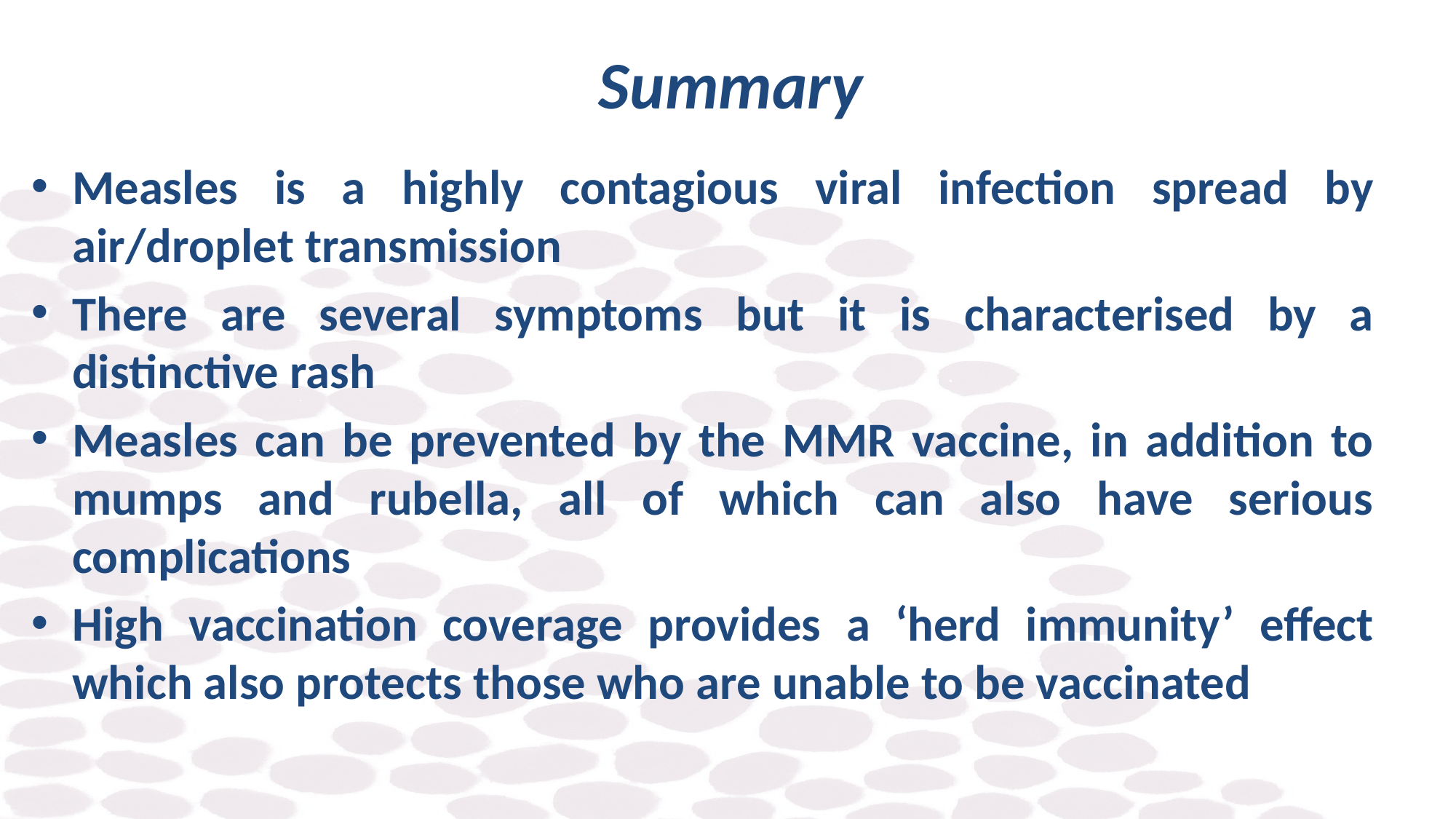

# Summary
Measles is a highly contagious viral infection spread by air/droplet transmission
There are several symptoms but it is characterised by a distinctive rash
Measles can be prevented by the MMR vaccine, in addition to mumps and rubella, all of which can also have serious complications
High vaccination coverage provides a ‘herd immunity’ effect which also protects those who are unable to be vaccinated
