# Supplementary material for: Impact of educational interventions on adolescent attitudes and knowledge regarding vaccination: A pilot study
Source: PLoS One. 2018 Jan 19;13(1):e0190984. doi: 10.1371/journal.pone.0190984 (PMC5774691; doi:10.1371/journal.pone.0190984)
Supplement: S1 Dataset — (DOCX) [file pone.0190984.s005.docx]

**S5: Attitudinal scores of main trial participants**

| **Digital Group (A)** | | | |  | **PowerPoint Group (B)** | | | |  | **Control Group (C)** | | | |
| --- | --- | --- | --- | --- | --- | --- | --- | --- | --- | --- | --- | --- | --- |
| # | Baseline | After  intervention | Follow Up |  | # | Baseline | After intervention | Follow Up |  | # | Baseline | After intervention | Follow Up |
| 1 | 35 | 33 | 33 |  | 1 | 32 | 34 | 37 |  | 1 | 36 | 34 | 34 |
| 2 | 35 | 35 | X |  | 2 | 35 | 33 | 31 |  | 2 | 33 | 29 | 38 |
| 3 | 33 | 33 | 32 |  | 3 | 28 | 32 | 31 |  | 3 | 30 | 30 | 33 |
| 4 | 30 | 31 | 29 |  | 4 | 29 | 33 | 34 |  | 4 | 32 | 36 | 32 |
| 5 | 31 | 31 | 33 |  | 5 | 30 | 32 | 33 |  | 5 | 30 | 34 | 35 |
| 6 | 30 | 29 | 31 |  | 6 | 35 | 36 | 36 |  | 6 | 27 | 35 | 32 |
| 7 | 36 | 31 | 28 |  | 7 | 36 | 35 | 36 |  | 7 | 31 | 26 | 33 |
| 8 | 31 | 36 | 33 |  | 8 | 33 | 35 | 34 |  | 8 | 27 | 30 | 35 |
| 9 | x | 30 | X |  | 9 | 29 | 30 | 36 |  | 9 | 36 | 30 | 38 |
| 10 | x | 31 | 34 |  | 10 | 39 | 38 | 38 |  | 10 | 28 | 27 | 31 |
| 11 | 32 | 35 | 34 |  | 11 | 35 | 37 | x |  | 11 | 29 | 30 | 33 |
| 12 | 30 | 31 | 31 |  | 12 | 35 | 36 | 32 |  | 12 | 34 | 32 | 38 |
| 13 | 30 | 32 | 32 |  | 13 | 30 | 33 | 32 |  | 13 | 32 | 33 | 33 |
| 14 | 31 | x | 31 |  | 14 | x | 29 | 35 |  | 14 | 34 | 32 | 33 |
| 15 | 31 | 32 | 29 |  | 15 | 30 | 35 | 35 |  | 15 | 34 | 33 | 32 |
| 16 | 34 | 34 | X |  | 16 | 30 | 33 | x |  | 16 | 30 | 26 | 33 |
| 17 | 32 | 37 | 30 |  | 17 | 33 | 35 | 36 |  |  |  |  |  |
| 18 | 32 | 31 | 34 |  | 18 | 35 | 34 | 29 |  |  |  |  |  |
| 19 | 28 | 36 | 35 |  | 19 | 32 | 32 | x |  |  |  |  |  |
| 20 | 27 | 27 | X |  | 20 | 32 | 31 | 32 |  |  |  |  |  |
| 21 | 29 | 30 | 36 |  | 21 | x | 32 | x |  |  |  |  |  |
| 22 | 30 | 34 | 29 |  |  |  |  |  |  |  |  |  |  |
| 23 | 35 | 38 | X |  |  |  |  |  |  |  |  |  |  |
| 24 | 30 | 33 | X |  |  |  |  |  |  |  |  |  |  |
| 25 | 30 | 34 | 32 |  |  |  |  |  |  |  |  |  |  |
| 26 | 32 | 34 | X |  |  |  |  |  |  |  |  |  |  |
|  |  |  |  |  |  |  |  |  |  |  |  |  |  |

**Change in attitudinal scores of main trial participants**

| Digital Group (A) | | | |  | PowerPoint Group (B) | | | |  | Control Group (C) | | | |
| --- | --- | --- | --- | --- | --- | --- | --- | --- | --- | --- | --- | --- | --- |
| # | Baseline | After intervention | Follow Up |  | # | Baseline | After intervention | Follow Up |  | # | Baseline | After intervention | Follow Up |
| 1 | 35 | - | 0 |  | 1 | 32 | + | + |  | 1 | 36 | - | 0 |
| 2 | x | x | X |  | 2 | 35 | - | - |  | 2 | 33 | - | + |
| 3 | 33 | 0 | - |  | 3 | 28 | + | - |  | 3 | 30 | 0 | + |
| 4 | 30 | + | - |  | 4 | 29 | + | + |  | 4 | 32 | + | - |
| 5 | 31 | 0 | + |  | 5 | 30 | + | + |  | 5 | 30 | + | + |
| 6 | 30 | - | + |  | 6 | 35 | + | 0 |  | 6 | 27 | + | - |
| 7 | 36 | - | - |  | 7 | 36 | - | + |  | 7 | 31 | - | + |
| 8 | 31 | + | - |  | 8 | 33 | + | - |  | 8 | 27 | + | + |
| 9 | x | x | X |  | 9 | 29 | + | + |  | 9 | 36 | - | + |
| 10 | x | x | x |  | 10 | 39 | - | 0 |  | 10 | 28 | - | + |
| 11 | 32 | + | - |  | 11 | x | x | x |  | 11 | 29 | + | + |
| 12 | 30 | + | 0 |  | 12 | 35 | + | - |  | 12 | 34 | - | + |
| 13 | 30 | + | 0 |  | 13 | 30 | + | - |  | 13 | 32 | + | 0 |
| 14 | x | x | x |  | 14 | x | x | x |  | 14 | 34 | - | + |
| 15 | 31 | + | - |  | 15 | 30 | + | 0 |  | 15 | 34 | - | - |
| 16 | 34 | 0 | X |  | 16 | x | x | x |  | 16 | 30 | - | + |
| 17 | 32 | + | - |  | 17 | 33 | + | + |  |  |  |  |  |
| 18 | 32 | - | + |  | 18 | 35 | - | - |  |  |  |  |  |
| 19 | 28 | + | - |  | 19 | x | x | x |  |  |  |  |  |
| 20 | x | x | X |  | 20 | 32 | - | + |  |  |  |  |  |
| 21 | 29 | + | + |  | 21 | x | x | x |  |  |  |  |  |
| 22 | 30 | + | - |  |  |  |  |  |  |  |  |  |  |
| 23 | x | x | X |  |  |  |  |  |  |  |  |  |  |
| 24 | x | x | X |  |  |  |  |  |  |  |  |  |  |
| 25 | 30 | + | - |  |  |  |  |  |  |  |  |  |  |
| 26 | x | x | X |  |  |  |  |  |  |  |  |  |  |
|  |  |  |  |  |  |  |  |  |  |  |  |  |  |
