# Supplementary material for: Impact of educational interventions on adolescent attitudes and knowledge regarding vaccination: A pilot study
Source: PLoS One. 2018 Jan 19;13(1):e0190984. doi: 10.1371/journal.pone.0190984 (PMC5774691; doi:10.1371/journal.pone.0190984)
Supplement: S2 Dataset — (DOCX) [file pone.0190984.s006.docx]

**S6: Information needs/personal choice dataset**

**Q1: More information about vaccinations should be given to me**

|  | Digital Group (A) (A) | | | Presentation Group (B) (B) | | | Control Group (C) (C) | | |
| --- | --- | --- | --- | --- | --- | --- | --- | --- | --- |
|  | Baseline | After intervention | Follow up | Baseline | After intervention | Follow up | Baseline | After intervention | Follow up |
| Strongly disagree | 2 | 2 | 1 | 0 | 0 | 0 | 1 | 0 | 0 |
| Disagree | 3 | 4 | 3 | 1 | 1 | 2 | 0 | 1 | 1 |
| Neither agree nor disagree | 6 | 7 | 3 | 5 | 8 | 7 | 4 | 7 | 6 |
| Agree | 12 | 11 | 10 | 12 | 9 | 7 | 11 | 8 | 8 |
| Strongly agree | 4 | 2 | 2 | 2 | 3 | 1 | 0 | 0 | 1 |

Q2: I know all I need to know about vaccination and how it works

|  | Digital Group (A) (A) | | | Presentation Group (B) | | | Control Group (C) | | |
| --- | --- | --- | --- | --- | --- | --- | --- | --- | --- |
|  | Baseline | After intervention | Follow up | Baseline | After intervention | Follow up | Baseline | After intervention | Follow up |
| Strongly disagree | 1 | 0 | 1 | 2 | 0 | 0 | 0 | 0 | 0 |
| Disagree | 13 | 6 | 9 | 7 | 5 | 3 | 2 | 4 | 2 |
| Neither agree nor disagree | 6 | 6 | 4 | 7 | 5 | 7 | 2 | 2 | 5 |
| Agree | 5 | 11 | 4 | 4 | 10 | 7 | 7 | 7 | 5 |
| Strongly agree | 1 | 3 | 1 | 0 | 1 | 0 | 5 | 3 | 4 |

Q3: Children should have more say than their parents should when it comes to their own vaccinations

|  | Digital Group (A) | | | Presentation Group (B) | | | Control Group (C) | | |
| --- | --- | --- | --- | --- | --- | --- | --- | --- | --- |
|  | Baseline | After intervention | Follow up | Baseline | After intervention | Follow up | Baseline | After intervention | Follow up |
| Strongly disagree | 3 | 3 | 1 | 4 | 4 | 2 | 4 | 1 | 3 |
| Disagree | 6 | 10 | 7 | 5 | 6 | 4 | 8 | 9 | 8 |
| Neither agree/disagree | 6 | 4 | 6 | 7 | 5 | 8 | 1 | 3 | 4 |
| Agree | 8 | 8 | 3 | 2 | 2 | 2 | 0 | 3 | 1 |
| Strongly agree | 4 | 1 | 2 | 2 | 4 | 1 | 3 | 0 | 0 |

Q4: Someone under 16 who is well informed should be able to choose to be (or not be) vaccinated without their parent’s consent

|  | Digital Group (A) | | | Presentation Group (B) | | | Control Group (C) | | |
| --- | --- | --- | --- | --- | --- | --- | --- | --- | --- |
|  | Baseline | After intervention | Follow up | Baseline | After intervention | Follow up | Baseline | After intervention | Follow up |
| Strongly disagree | 3 | 1 | 0 | 0 | 1 | 1 | 3 | 2 | 1 |
| Disagree | 9 | 12 | 4 | 5 | 5 | 2 | 2 | 7 | 2 |
| Neither agree/disagree | 6 | 2 | 3 | 7 | 6 | 6 | 5 | 6 | 5 |
| Agree | 6 | 8 | 11 | 4 | 7 | 5 | 5 | 1 | 6 |
| Strongly agree | 3 | 3 | 1 | 4 | 2 | 3 | 1 | 0 | 2 |

Q5: Doctors, not parents or their children, should have the final say about if a child is vaccinated

|  | Digital Group (A) | | | Presentation Group (B) | | | Control Group (C) | | |
| --- | --- | --- | --- | --- | --- | --- | --- | --- | --- |
|  | Baseline | After intervention | Follow up | Baseline | After intervention | Follow up | Baseline | After intervention | Follow up |
| Strongly disagree | 4 | 4 | 1 | 1 | 1 | 2 | 1 | 0 | 1 |
| Disagree | 8 | 12 | 7 | 7 | 2 | 3 | 7 | 11 | 7 |
| Neither agree/disagree | 11 | 4 | 4 | 6 | 6 | 6 | 5 | 3 | 1 |
| Agree | 3 | 4 | 4 | 5 | 8 | 3 | 2 | 1 | 7 |
| Strongly agree | 1 | 2 | 3 | 1 | 4 | 3 | 1 | 1 | 0 |

Q6: It is nobody else’s business if I am vaccinated

|  | Digital Group (A) | | | Presentation Group (B) | | | Control Group (C) | | |
| --- | --- | --- | --- | --- | --- | --- | --- | --- | --- |
|  | Baseline | After intervention | Follow up | Baseline | After intervention | Follow up | Baseline | After intervention | Follow up |
| Strongly disagree | 2 | 2 | 0 | 1 | 3 | 4 | 1 | 2 | 1 |
| Disagree | 3 | 4 | 3 | 0 | 3 | 4 | 2 | 3 | 2 |
| Neither agree/disagree | 11 | 8 | 6 | 6 | 7 | 5 | 8 | 7 | 9 |
| Agree | 7 | 9 | 9 | 11 | 6 | 3 | 3 | 2 | 2 |
| Strongly agree | 4 | 3 | 1 | 2 | 2 | 1 | 2 | 2 | 2 |
